# *Xszen*FHal, a novel tryptophan 5-halogenase from *Xenorhabdus szentirmaii*

**DOI:** 10.1186/s13568-019-0898-y

**Published:** 2019-10-31

**Authors:** Jérémy Domergue, Diane Erdmann, Aurélie Fossey-Jouenne, Jean-Louis Petit, Adrien Debard, Véronique de Berardinis, Carine Vergne-Vaxelaire, Anne Zaparucha

**Affiliations:** Génomique métabolique, Genoscope, Institut François Jacob, CEA, CNRS, Univ Evry, Université Paris-Saclay, 91057 Evry, France

**Keywords:** Flavin-dependent halogenases, Biocatalysis, Electrophilic halogenation, Regioselectivity, Indole derivatives

## Abstract

Flavin-dependent halogenases (FHals) catalyse the halogenation of electron-rich substrates, mainly aromatics. Halogenated compounds have many applications, as pharmaceutical, agrochemicals or as starting materials for the synthesis of complex molecules. By exploring the sequenced bacterial diversity, we discovered and characterized *Xszen*FHal, a novel FHal from *Xenorhabdus szentirmaii*, a symbiotic bacterium of entomopathogenic nematode. The substrate scope of *Xszen*FHal was examined and revealed activities towards tryptophan, indole and indole derivatives, leading to the formation of the corresponding 5-chloro products. *Xszen*FHal makes a valuable addition to the panel of flavin-dependent halogenases already discovered and enriches the potential for biotechnology applications by allowing access to 5-halogenated indole derivatives.

## Introduction

Halogenated compounds are a major category of molecules in organic chemistry as finished products with applications in pharmacy and agrochemistry, but also as intermediates in metal-catalyzed coupling reactions. Halogenations of aromatic cycles are still an issue in conventional synthesis, due to low reactivity and medium selectivity, which makes this route a real challenge on industrial scale. As a possible answer, the use of enzyme catalysed halogenation reaction is particularly interesting in a greener context (Fraley and Sherman 2018; Gkotsi et al. 2018; Latham et al. 2018; Schnepel and Sewald 2017; Weichold et al. 2016). Flavin-dependent halogenases (FHals) are halogenating enzymes catalyzing electrophilic halogenation of electron-rich aromatic and heteroaromatic compounds. They use harmless alkali halides and are highly regioselective; therefore they represent a promising alternative to conventional halogenation methods (Maddox et al. 2015; Mitchell et al. 1979; Prakash et al. 2004; Samanta and Yamamoto 2015; Schröder et al. 2012; van Pee 2012).

Flavin-dependent halogenases constitute a small enzyme family belonging to the superfamily of flavin-dependent monooxygenases and only few enzymes are described which restricts their application in synthesis (Gkotsi et al. 2018; Latham et al. 2018; Mascotti et al. 2016). The halogenase PrnA was the first FHal to be identified. It catalyses the C7-regioselective halogenation of tryptophan in the biosynthetic pathway of pyrrolnitrin, an antifungal metabolite produced by *Pseudomonas fluorescens*. The purification and characterization of this enzyme reveal that its activity was Flavin Adenine Dinucleotide (FAD) dependent and requires a NAD(P)H flavin reductase for FADH_2_ supply (Hohaus et al. 1997; Keller et al. 2000). Structural elucidation of PrnA by X-ray crystallography allowed to propose a mechanism for the electrophilic regioselective halogenation (Dong et al. 2005; Keller et al. 2000). FHals are active towards electron-rich substrates such as indole and phenol derivatives, but also β-keto carbonyl moieties (Jungmann et al. 2015; Podzelinska et al. 2010; van Pee and Patallo 2006).

Many of these enzymes are incorporated in the enzymatic machinery of polyketide or non-ribosomal peptide synthases and are, therefore, not readily available for biocatalytic purposes (Buedenbender et al. 2009; Chiu et al. 2001; Dorrestein et al. 2005; Heide et al. 2008; Hornung et al. 2007; Jungmann et al. 2015; Lin et al. 2007; Podzelinska et al. 2010; Rachid et al. 2006; Son et al. 2017; Yu et al. 2002). To access new FHals and thus broaden the range of possibilities, one option is to explore the extraordinary amount of available genomic resources (Zaparucha et al. 2018). As halogenated secondary metabolites are widespread compounds, halogenases can be found in various organisms and there are still a lot to discover (Neubauer et al. 2018; Smith et al. 2013; Son et al. 2017). In this study, we explored the sequenced bacterial diversity in search of novel flavin-dependent halogenases in order to find enzymes active towards aromatic electron-rich substrates.

## Materials and methods

### Chemicals and materials

All reagents were purchased from commercial sources and used without additional purification. d,l-tryptophan, d-tryptophan, l-tryptophan, d,l-tyrosine, 2-amino benzoic acid, 4-amino benzoic acid, 2-amino-5-chloro benzoic acid, 2-amino-3-chloro benzoic acid, 5-hydroxy-l-tryptophan, tryptamine, 3-indoleacetonitrile, 3-(2-hydroxyethyl)indole, indole, 6-chloro indole, indole-3-pyruvic acid, indole-3-acrylic acid, indole-3-butyric acid, glucose, NADH and FAD were purchased from Sigma Aldrich (MilliporeSigma, StLouis, USA). Indole-3-acetic acid was purchased from Acros Organics (Fisher Scientific SAS, Illkirch, France). Indole-3-propionic acid was purchased from Alfa Aesar (Thermo Fisher (Kandel) GmbH, Karlsruhe, Germany). 5-Chloro-tryptophan and 5-chloro tryptamine were purchased from Chem Impex International Inc. (Wood Dale, USA). 5-Chloro-3-(2-hydroxyethyl)indole and 5-chloro-indole-3-acetamide were purchased from Oxchem Corporation (Wood Dale, USA). 5-Bromo-tryptophan, 5-chloro-indole-3-acetonitrile and 5-chloroindole were purchased from Enamine (LLC Monmouth Jct, USA). Indole-3-acetamide was purchased from TCI Europe (Zwijndrecht, Belgium). 7-Chloro tryptophan was a generous gift from Dr. Eugenio P. Patallo (Institut für Biochemie, TU Dresden, 01062 Dresden, Germany) (Zhu et al. 2009). Regeneration enzyme glucose dehydrogenase 105 (GDH-105) was given by Codexis (Redwood City, USA). For the screening, filter plates were used (Acroprep Advance 96 Filter Plate, 0.2 μm GHP, Pall). For reactions in Eppendorf tubes, simple filters were used (0.2 μm, PTFE, Merck Millipore). Oligonucleotides were from Sigma-Aldrich (MilliporeSigma, StLouis, USA). *E*. *coli* strains BL21-CodonPlus (DE3)-RIPL were from Agilent technologies (Santa Clara, USA). Enzymatic spectrophotometric screenings in microplates were performed on a SpectraMax Plus384 (Molecular Devices, Sunnyvale, USA). Cell-free extracts in 96-microwell plates were heated using a tested thermocycler GeneAmpTM PCR system 9700 (Applied Biosystem, Foster City, USA). NMR spectra were recorded on a Bruker (Bruker, Billerica, USA) 600 MHz spectrometer (Evry University, France) for ^1^H and ^13^C experiments. Chemical shifts are expressed in ppm and referenced with residual solvent signals. Coupling constant values (*J*) are given in hertz. UHPLC-UV analyses were performed on a UHPLC U3000 RS 1034 bar (Thermo Fisher Scientific, Waltham, USA) equipped with a thermostated Column Compartment Rapid Separation (TCC-3000RS) and a diode array detector DAD3000. UHPLC-HRMS analyses were performed on a UHPLC U3000 RS 1034 bar (Thermo Fisher Scientific, Waltham, USA) equipped with a Thermostated Column Compartment Rapid Separation (TCC-3000RS) coupled to ultra-high resolution Orbitrap Elite hybrid mass spectrometer (Thermo Fisher Scientific, Waltham, USA) equipped with electrospray ionization (ESI) source.

### Selection of candidate enzymes

A three-step process was conducted for searching new FHals (Vergne-Vaxelaire et al. 2013). First of all, a reference set was created by collecting known proteins from the literature (Additional file 1: Table S1 and Figure S1). Second, this set was used for protein-versus protein alignments, using the BL2 option (BLAST allowing gaps) and a BLOSUM62 score matrix against UniprotKB and the metagenome from Genoscope using low stringency parameters (> 30% of identity, on 80% of the length) resulting in the selection of 6574 candidate enzymes. Third, to minimize number of candidates, protein sequences were clusterized (80% of similarities) to create putative isofunctional groups using traditional all-against-all normalized BLASTP scores calculated by the LASSAP suite and a single-linkage algorithm. A representative candidate was then chosen, one by cluster, if its corresponding bacterial strain was available in the Genoscope genomic collection. All the sequences too divergent to be included in the clustering were chosen if the corresponding genome was available in the Genoscope genomic collection. Missing strains were purchased from DSMZ when available. Representatives of each cluster were cloned and engaged in screening processes.

### Cloning, expression and purification

Primers were chosen and genes were cloned with a histidine tag in *N*-terminal part in a pET22b(+) (Novagen) modified for ligation independent cloning as already described (Bastard et al. 2014). All primers and strains of the selected FHals are listed in Additional file 1: Table S2 and named from FHal1 to FHal148. All the strains along with their identifiers were purchased from DSMZ, CIP or ATCC collections. When DNA samples corresponding to the gene encoding the selected enzyme was not available, PCR was performed on the DNA of another strain from the same species as noted in Additional file 1: Table S2. All sequences were verified. Each expression plasmid was transformed into *Escherichia coli* BL21-CodonPlus (DE3)-RIPL. Cell culture, induction of protein production and cell lysis were conducted as previously published (Bastard et al. 2014). Selected enzymes from the screening (FHal13, FHal16, FHal35, FHal46, FHal57, FHal106) were then purified by loading the clear crude cell extract from 100 mL of culture onto a Ni–NTA column (QIAGEN), according to the manufacturer’s instructions. The elution buffer was 50 mM phosphate (pH 7.5), 50 mM NaCl, 250 mM imidazole and 10% glycerol. The flavin reductase from *E. coli* strain K12 (*K12*Fre) (UniProtKB ID: P0AEN1) was chosen as universal reductase for all FHAls. *K12*Fre was cloned, overexpressed and purified following the same protocol. Large scale protein purification was conducted from a 2 × 500 mL culture and was performed using a preparative chromatography system (Äkta Pure; GE Healthcare Life Sciences). A fully automated two-step method was set up in which a His Trap FF 5-mL (GE Healthcare Life Sciences) column was used in the first purification step. The eluted peak was redirected on a HiLoad 16/600 Superdex 200-pg size exclusion column (GE Healthcare Life Sciences) and collected in 50 mM phosphate (pH 7.5), 50 mM NaCl, 1 mM DTT and 10% glycerol. Purified enzymes were stored at − 80 °C. The samples were analyzed by SDS-PAGE using the NuPAGE system. Protein concentration was determined by the Bradford method, with bovine serum albumin as the standard (Bio-Rad).

### Enzymatic screening assay on aromatic electron-rich substrates

**Fig. 1:**
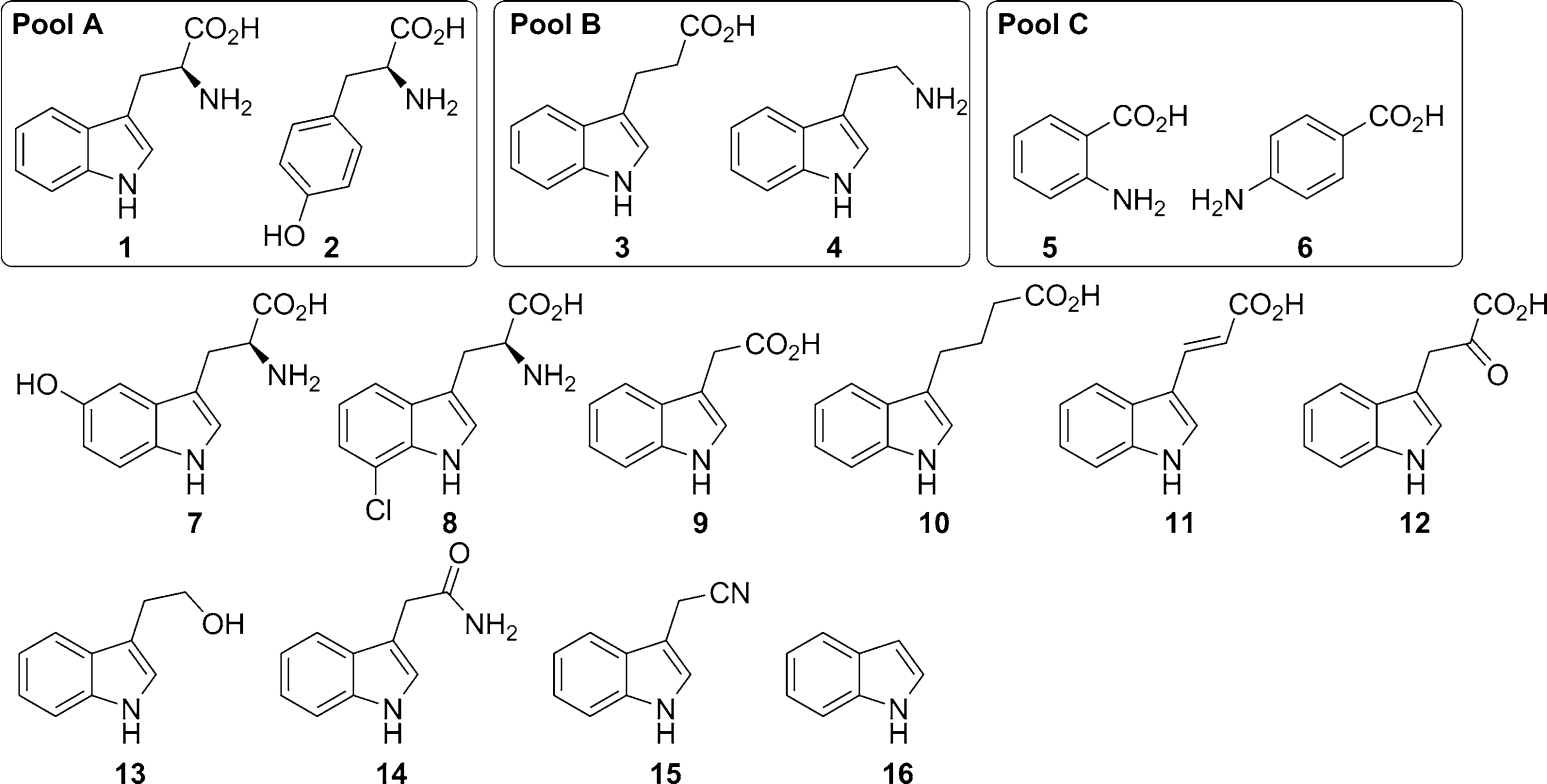
Substrate structures

The candidate FHals were screened as cell-free extracts in 96-microwell plates. Cell-free extracts were stored at − 80 °C, and thawed out on ice before use. The enzymatic reactions were carried out on three pools, A–B–C, composed of two substrates (vide infra) (final concentration 0.5 mM each), NaCl (20 mM), NADH (0.5 mM), FAD (1 μM), cell-free extract (10 μL), isopropanol (5 μL), phosphate buffer (10 mM, pH = 7.4) in a final volume of 100 μL at RT for 24 h. TFA (1 μL) was then added in each well, followed by 100 μL of water (Fig. 1). The microplates were centrifuged at 6000*g* for 10 min and the supernatants were filtrated in new microplates using filter plates (Acroprep Advance 96 Filter Plate, 0.2 µm GHP, Pall). A volume of 2 μL was then injected on UHPLC according to the conditions described below. Hits were determined by comparison with the reaction with cell-free extract without overexpressed protein. UHPLC analyses were conducted on an Accucore PFP (Thermo Scientific) column (50 * 2.1 mm, 2.6 μm) with eluents A (H_2_O + 0.1% formic acid) and B (CH_3_CN). Halogenated products were identified by UHPLC by comparison on their retention time with the ones of halogenated standards when available, and by mass spectroscopy. Pool A, tryptophan (**1**) and tyrosine (**2**): linear gradient (ratio A/B 99/1 during 1 min, then 99/1 to 85/15 in 2.5 min, then 85/15 during 2 min, then 85/15 to 50/50 in 1 min), flow 0.4 mL/min, λ = 276 nm. Pool B, tryptamine (**3**) and indol-3-acetic acid (**4**): linear gradient (ratio A/B 80/20 to 40/60 in 3 min, then 40/60 during 1 min), flow 0.4 mL/min, λ = 290 nm. Pool C, 2-aminobenzoic acid (**5**) and 4-aminobenzoic acid (**6**): linear gradient (ratio A/B 95/5 during 0.5 min, then 95/5 to 70/30 during 1 min, then 70/30 to 50/50 during 1.5 min, then 50/50 during 1 min), flow 0.4 mL/min, λ = 290 nm and λ = 330 nm.

### Characterization of *Xszen*FHal (FHal16) from *Xenorhabdus szentirmaii* DSM 16338

All specific activities and kinetic parameters were determined from duplicate experiments. All reactions were performed on purified *Xszen*FHal (Uniprot ID: W1J423) at 25 °C in 100 μL scale and monitored by UHPLC-UV (Additional file 1: Figure S2).

i. Standard conditions. The assay mixture comprised 1 mM substrate, 1 mg/mL *Xszen*FHal, 0.22 mg/mL *K12*Fre, 20 mM NaCl, 3 mM glucose, 3 U/mL GDH-105, 0.05 mM NADH, 1 μM FAD in phosphate buffer 10 mM pH 7.4. The reaction was stirred at 500 rpm for 24 h then 1 μM TFA and 70 μL isopropanol were added. After centrifugation at 6000 rpm for 10 min, the supernatant was filtered over PVDF membrane filter 0.2 μm and analyzed by UHPLC-UV (see below, UHPLC conditions for microscale reactions).
ii. Halide tolerance. The assay mixture comprised 1 mM l-tryptophan, 0.25 mg/mL *Xszen*FHal, 0.055 mg/mL *K12*Fre, 20 mM NaX, 3 mM glucose, 1 U/mL GDH, 0.025 mM NADH, 1 μM FAD in phosphate buffer 10 mM pH 7.4. The reaction was stirred at 500 rpm for 24 h then 1 μM TFA and 70 μL isopropanol were added. After centrifugation at 6000 rpm for 10 min, the supernatant was filtered over PVDF membrane filter 0.2 μM and analyzed by UHPLC-UV using conditions A.
iii. Specific activity towards l-tryptophan (**1**). The assay mixture comprised 1 mM substrate, 0.2 mg/mL *Xszen*FHal, 0.022 mg/mL *K12*Fre, 20 mM NaX, 3 mM glucose, 1 U/mL GDH, 0.025 mM NADH, 1 μM FAD in phosphate buffer 10 mM pH 7.4. The reaction was stirred at 500 rpm then 1 μM TFA and 70 μL isopropanol were added. After centrifugation at 6000 rpm for 10 min, the supernatant was filtered over PVDF membrane filter 0.2 μm and analyzed by UHPLC-UV using conditions A.
iv. Specific activities toward various substrates (Fig. 1). The typical assay mixture comprised 1 mM substrate, 0.0.25 mg/mL *K12*Fre, 20 mM NaCl, 3 mM glucose, 3 U/mL GDH, 0.05 mM NADH, 1 μM FAD in phosphate buffer 10 mM pH 7.4 and *Xszen*FHal at the indicated concentration. Tested substrates were: 7-Cl-tryptophan (**8**); indole-3-ethanol (**13**); indole-3-acetamide (**14**); indole-3-acetonitrile (**15**). The reaction was stirred at 500 rpm then 1 μM TFA and 70 μL isopropanol were added. After centrifugation at 6000 rpm for 10 min, the supernatant was filtered over PVDF membrane filter 0.2 μm and analyzed by UHPLC-UV. Enzyme concentration and monitoring time have been optimized to fall within the linear range of enzyme activity. The specific activities were calculated based on the quantity of product formed over the defined time period, determined by calibration curves with commercial standards.
v. Determination of kinetic parameters. The assay mixture comprised 1 μM *Xszen*FHal, 0.1 μM *K12*Fre, 20 mM NaCl, 0.05 mM NADH, 0.01 mM FAD in phosphate buffer 10 mM pH 7.5. The following tryptophan concentrations were used: 5 μM; 20 μM; 100 μM; 250 μM; 500 μM; 1000 μM; 1500 μM. The reaction was stirred at 500 rpm then 1 μM TFA and 70 μL isopropanol were added. After centrifugation at 6000 rpm for 10 min, the supernatant was filtered over PVDF membrane filter 0.2 μm and analyzed by UHPLC-UV. Enzyme concentration and monitoring time have been optimized to fall within the linear range of enzyme activity. Kinetic parameters were determined by fitting initial rate data to the Michaelis–Menten equation (Additional file 1: Figure S6).

### UHPLC conditions for microscale reactions

UHPLC-UV conditions for substrates (**1**)-(**3**–**4**)-(**7**–**16**): UPLC analyses were conducted on an Accucore PFP (Thermo Scientific) column (50 * 2.1 mm, 2.6 μm), eluents A (H_2_O + 0.1% formic acid) and B (CH_3_CN) with the following conditions:

Conditions A for substrate (**1**): linear gradient (ratio A/B 99/1 during 1 min, then 99/1 to 85/15 in 2.5 min, 85/15 for 2.5 min then 85/15 to 50/50 in 1 min), flow 0.4 mL/min, λ = 276 nm.

Conditions B for substrate (**4**): linear gradient (ratio A/B 80/20 to 40/60 in 3 min, 40/60 for 2.5 min then 40/60 to 20/80 in 1.5 min, 20/80 for 0.5 min), flow 0.4 mL/min, λ = 220, 245, 280 and 330 nm.

Conditions C for substrate (**7**): linear gradient (ratio A/B 95/5 for 0.5 min, 95/5 to 70/30 in 1 min, 70/30 to 30/70 in 2 min, 30/70 for 1 min), flow 0.4 mL/min, λ = 230, 275, 280 and 330 nm.

Conditions D for substrates (**8**–**9**)-(**13**–**14**)-(**16**): linear gradient (ratio A/B 80/20 to 10/90 in 3.5 min, then 10/90 for 1 min), flow 0.4 mL/min, λ = 220 and 280 nm.

Conditions E for substrates (**3**)-(**10**–**12**)-(**15**): linear gradient (ratio A/B 60/40 to 10/90 in 2.5 min, then 10/90 for 2 min), flow 0.4 mL/min, λ = 220, 230, 280 and 330 nm.

### Synthesis of 5,7-dichloro-l-tryptophan (17)

The synthesis of 5,7-dichloro-l-tryptophan (**17**) was accomplished following a procedure described by Heemstra and coworkers for the synthesis of 6,7-dichloro-l-tryptophan (Heemstra and Walsh 2008) (Additional file 1: Scheme S1).

5,7-Dichloro l-tryptophan. RMN ^1^H (CD_3_OD, 600 mHz): δ 7.68 (s, 1H, H-4); 7.39 (s, 1H, H-2); 7.26 (s, 1H, H-6); 3.45 (m, 1H, H-11); 3.24 to 3.07 (m, 2H, H-10). RMN ^13^C (CD_3_OD, 125 mHz): δ 176.0 (C-12); 132.7 (C8); 130.8 (C-9); 128.3 (C-2); 123.9 (C-7); 120.8 (C-6); 118.3 (C-4); 117.3 (C-5); 112.0 (C-3); 55.5 (C-11); 27.6 (C-10). HRMS (ESI^+^): [M+H]^+^ m/z calcd for C_11_H_10_Cl_2_N_2_O_2_, 273.01921; found 273.01940.

## Results

### Halogenase screening

The protein sequences of 22 known FHals were used in a sequence-driven approach to build a collection of halogenase similar enzymes (Vergne-Vaxelaire et al. 2013). 6574 proteins sharing at least 30% identity over at least 80% of the length with the enzymes from reference set were collected. Proteins have been clustered based on sequence identity (≥ 80%) and one representative per cluster, for which genomic DNA was available in the Genoscope strain collection, was chosen. From the 417 candidates selected to represent sequence diversity, 148 genes were successfully cloned in an expression vector (Additional file 1: Table S2). After overexpression of recombinant genes in *Escherichia coli* strain BL21, cells were lyzed and the proteins were quantified. No extra flavin reductase (Fre) was added as *E. coli* contains naturally occurring flavin reductases. The candidate enzymes were screened as cell-free extracts against six aromatic electron-rich substrates grouped in three pools of two compounds (Fig. 1). Halogenase activity was assayed by UHPLC-UV. Only four enzymes were found to be very weakly or moderately active compared to a blank reaction, FHal13 (UniProtKB ID: Q4J6K2), FHal35 (UniProtKB ID: B7J6K4) and FHal46 (UniProtKB ID: C7PAY7) towards substrates from pool C, and FHal16 (UniProtKB ID: W1J423) towards substrates from pools A and C. The four enzymes cloned with a polyhistidine tag were purified by nickel affinity chromatography for further studies and their activities were verified on single substrate. In our reaction conditions, only FHal16 confirmed to have a halogenase activity and was found to be active towards tryptophan. FHal16, from *X. szentirmaii* DSM16338, has been named *Xszen*FHal.

### Characterization of *Xszen*FHal

*Xszen*FHal, from *X. szentirmaii* DSM16338, shares ~ 60% sequence identity with PyrH (Uniprot ID: A4D0H5), its closest homolog in the reference set (Gualtieri et al. 2014). PyrH is involved in the pyrroindomycin B biosynthesis, an antibiotic compound produced by *Streptomyces rugosporus* LL-42D005, where it catalyzes the C5 chlorination of tryptophan (Zehner et al. 2005) (Additional file 1: Figures S3 and S4). *X. szentirmaii* is a symbiotic bacterium of entomopathogenic nematode (Lengyel et al. 2005). It is known to produce various secondary metabolites, xenofuranones A and B, two phenylpyruvate dimers, szentiamide, a depsipeptide and fabclavines, PK-NRP-polyamine hybrids (Brachmann et al. 2006; Ohlendorf et al. 2011; Wenski et al. 2019) (Additional file 1: Figure S4). From all these metabolites, only szentiamide has a tryptophan moiety but it is non chlorinated. The analysis of the genomic context of gene encoding *Xszen*FHal did not reveal any clear metabolic role. Its native substrate is so difficult to hypothesize.

**Scheme 1:**
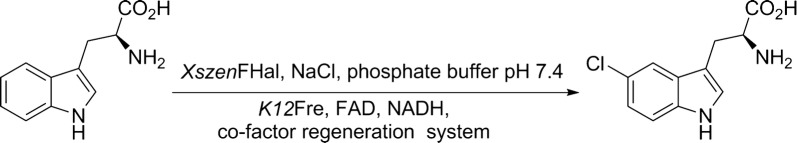
Chlorination of l-tryptophan catalyzed by *Xszen*FHal

**Fig. 2:**
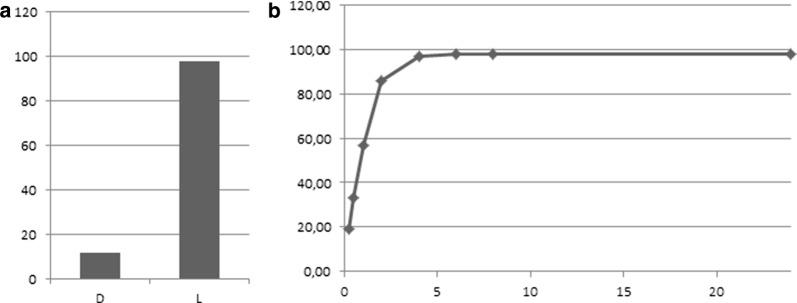
**a** d- and l-tryptophan conversions by *Xszen*FHal with NaCl over 24 h. **b** Time course of l-tryptophan conversion by *Xszen*FHal with NaCl over 24 h
ii. Substrate activity profile
  *Xszen*FHal was screened against various indole derivatives (**7**–**16**) (Fig. 1). The enzyme exhibited broad scope, seven of the 11 substrates being halogenated with low (entries 2 and 8) to medium (entries 10 and 11) conversions over 24 h and up to good (entries 10 and 12) conversions over 48 h (Table 1). None of the indole derivatives substituted with carboxylic acid functions was found to be substrate (entries 4–7). It is worthy to note that *Xszen*FHal was active towards all the simple indole derivatives with moderate to good conversions (entries 9–11) except in the case of the tryptamine for which a low conversion was obtained (entry 8).

Purified *Xszen*FHal was obtained on large scale using fully automated two-step method combining nickel affinity and size exclusion chromatography. To evaluate the substrate range of *Xszen*FHal, we screened it against various substrates. Because the preliminary screening showed that *Xszen*FHal was only active towards tryptophan and had no activity towards tyrosine and aniline derivatives, we focused our study on indole derivatives (Fig. 1). Purified *Xszen*FHal and *K12*Fre were used to optimize the reaction conditions of tryptophan chlorination on small scale including glucose dehydrogenase as cofactor regeneration system (Payne et al. 2013; van der Donk and Zhao 2003; Yeh et al. 2005) (Scheme 1). Standard conditions were then used since no variation in parameters (NADH, FAD, NaCl) has allowed any improvement.

i. Stereospecificity
  *Xszen*FHal showed a clear stereopreference for the l-enantiomer with a 98% conversion into the chloro derivative within 6 h, while the conversion only reached 12% for the d-enantiomer over 24 h (Fig. 2).

**Table 1:**
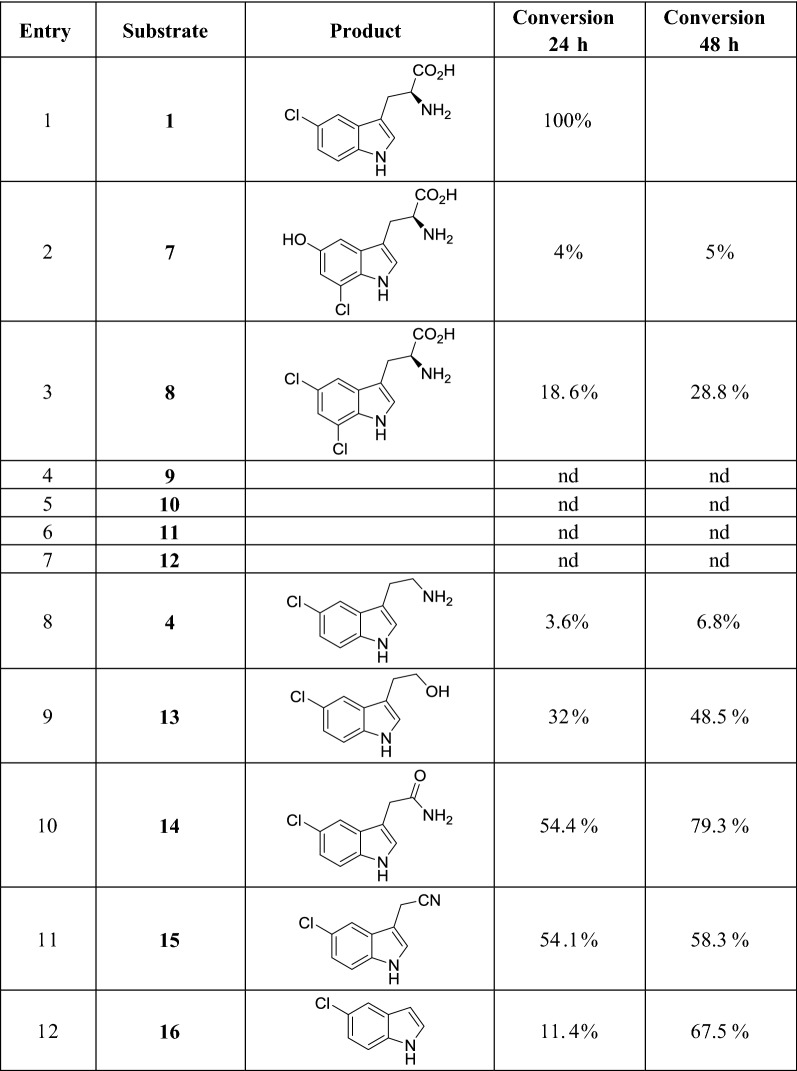
Conversion of various substrates by *Xszen*FHal with NaCl
iii. Regioselectivity
  Chlorination by *Xszen*FHal is C5 regioselective as demonstrated by comparison on UHPLC-UV analysis of the products with the UHPLC chromatograms of halogenated standards (Additional file 1: Figure S2).
iv. Halide tolerance
  We tested *Xszen*FHal for its ability to catalyze bromination and iodination of l-tryptophan, in addition to chlorination. Under standard conditions, *Xszen*FHal fully converted l-tryptophan to the 5-chloro derivative while bromination under the same conditions led to a 64% conversion (Additional file 1: Figure S5). No conversion was observed with NaI.

### Kinetic constants

i. Specific activities
  Specific activities were determined with l-tryptophan under NaCl and NaBr conditions and under NaCl conditions for the other substrates (Table 2). Specific activities were found moderate with l-tryptophan (entries 1–2) with ca. 2 times lower activity with NaBr (1.076 mU/mg) compared to NaCl (2.385 mU/mg). For indole derivatives, the specific activities were significantly lower, with values at least 10 times lower (entries 3–6).

**Table 2:**
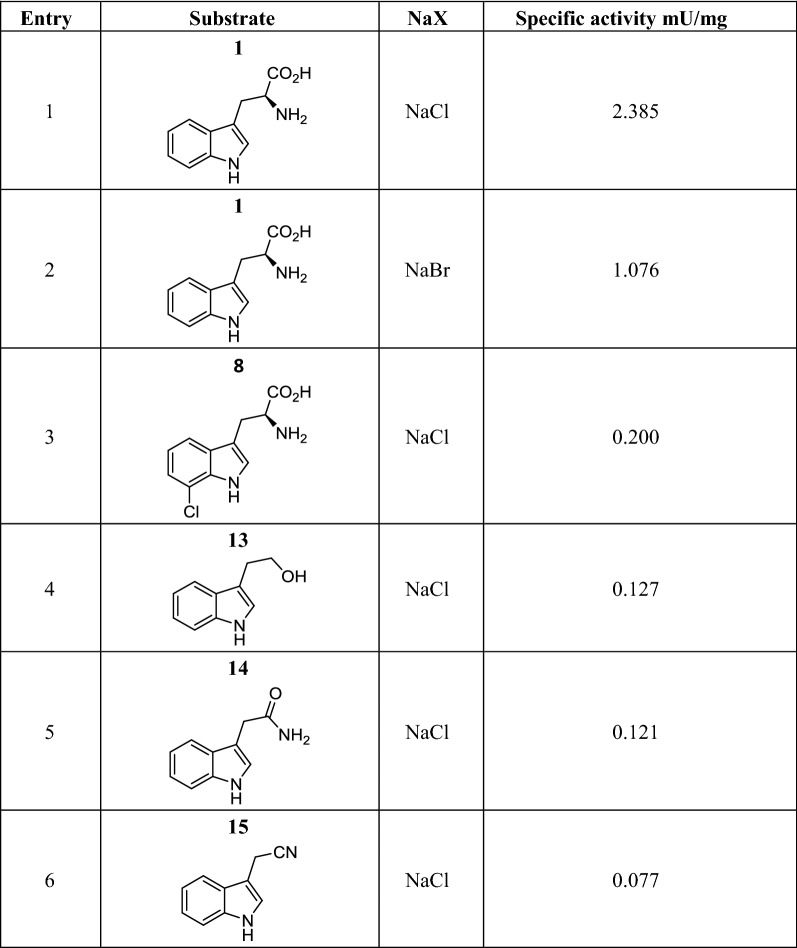
Specific activities of *Xszen*FHal on various substrates

One unit (U) of enzyme activity was defined as the amount of enzyme that catalyzed the conversion of 1 µM of substrate per minute
ii. Kinetic constants with l-tryptophan
  Under NaCl conditions, the kinetic parameters were measured with respective values of V_m_ 4.44 μM min^−1^ (88.8 U/mg enzyme); *K*_m_ 58.22 μM; *k*_cat_ 4.44 min^−1^ which results in a catalytic efficiency of *k*_cat_/*K*_m_ 0.076 min^−1^.M^−1^ (Additional file 1: Figure S6).

## Discussion

In this work, we present the discovery and characterization of *Xszen*FHal, a novel 5-regioselective tryptophan halogenase belonging to the flavin-dependent halogenase family. Only one tryptophan 5-halogenase active towards isolated amino acid has been characterized so far, PyrH (UniProtKB ID A4D0H5) from *Streptomyces rugosporus*, but without study of its substrate range (Zehner et al. 2005). Recently, two other 5-regioselective tryptophan halogenases have been identified in biosynthetic pathways but they are implied in tailoring modifications of metabolites and are active towards tethered peptides. MibH (UniProtKB ID: W2EQU4) is responsible of the 5-chlorination of tryptophan in lantibiotic NAI-107, a ribosomally synthesized and post-translationally modified peptide (RiPP) produced by the actinomycete *Microbispora* sp. 107891, and Ulm24 (UniProtKB ID: A0A2D3E318) was identified in the genome of *Streptomyces* sp. KCB13F003 which produced ulleungmycins A and B, two chlorinated non-ribosomal peptides (NRP) (Ortega et al. 2017; Son et al. 2017). *Xszen*FHal shares nearly 60% identity with PyrH and Ulm24, and only 30% identity with MibH (Additional file 1: Figure S7).

Similar to tryptophan 5-, 6- and 7-halogenases, *Xszen*FHal exhibits residual activity towards substrates in addition to tryptophan (Andorfer et al. 2017; Frese et al. 2014; Menon et al. 2016; Neubauer et al. 2018; Smith et al. 2017). Nevertheless, as the examination of the metabolic context of the gene encoding for *Xszen*FHal did not provide any indication, there is no formal evidence that tryptophan is the metabolic substrate of *Xszen*FHal. However, *Xszen*FHal kinetic data are consistent to the ones of halogenases for which tryptophan is the metabolic substrate (Additional file 1: Table S3) (Dong et al. 2005; Heemstra and Walsh 2008; Luhavaya et al. 2019; Yeh et al. 2005; Zeng and Zhan 2011; Zhu et al. 2009). When compared to PyrH, the other 5-regioselective tryptophan halogenase, *Xszen*FHal exhibits kinetic values fairly similar with *k*_cat_ of 4.44 min^−1^ for *Xszen*FHal and 3.56 min^−1^ for PyrH, and substrate binding value, *K*_m_ 58.22 μM for *Xszen*FHal and 109 for PyrH, resulting in a higher catalytic efficiency for *Xszen*FHal, *k*_cat_/*K*_m_ 0.076 min^−1^ M^−1^ for *Xszen*FHal compared to 0.033 min^−1^ for PyrH (Zhu et al. 2009). The specific activity values of *Xszen*FHal on the different substrates show significant differences, with value at least 10 times higher for tryptophan, indicating that it is the best substrate within the tested ones. Tryptophan (**1**) and 7-chloro tryptophan (**8**) were halogenated with a 5-regioselectivity, while the 5-hydroxy tryptophan (**7**) was halogenated with a 7-regioselectivity, which corresponds to the other activated position. In our conditions, the conversion was total for tryptophan in 24 h, and much lower for substrates (**7**) and (**8**) (Table 1). Very interestingly, *Xszen*FHal exhibited activity towards indole (**16**) and 3-substituted derivatives (**4**; **13**-**15**) (Tables 1, 2). These results are promising for synthetic applications. Indeed, under conventional chemistry conditions, no 5-regioselective chlorination of simple indole derivatives is possible except in the case of tryptamine (**4**) (Somei et al. 1995; Hasegawa et al. 1999); for substrates (**13–15**) the 5-regioselective chlorination can occur only when the indole moiety is part of a more complex molecule (Bell and Stump Craig; Chatterjee et al.; Clerin et al.; Laronze et al. 2005); regarding simple indole (**16**), the direct chlorination by conventional synthetic methods undergoes to the formation of 5,7-dichloro indole (2000; Vennemann et al.). In addition, halogenated-5 indole and derivatives could be easily modified by metal-catalyzed coupling reactions to produce various compounds (Batail et al. 2011). It is worthy to note that combination of biocatalysis and palladium catalysis gave access to new C–H activation transformations than cannot be achieved by single catalysis (Durak et al. 2016; Latham et al. 2016; Roy et al. 2010).

Our studies led to the identification of *Xszen*FHal, a novel tryptophan 5-halogenase from *X. szentirmaii*. Study of *Xszen*FHal substrate scope revealed that, in addition to tryptophan, *Xszen*FHal is also active towards substituted tryptophan, indole and indole derivatives. It thus completes the range of regioselectivities achievable in biocatalysis by allowing the synthesis of 5-halogenated tryptophan, indole and indole derivatives. These results show the potential of *Xszen*FHal for synthetic applications.

## Supplementary information

**Additional file 1: Scheme S1.** Synthesis of 5,7-dichloro-L-tryptophan (**17**). **Table S1.** Enzymes used for the reference set. **Table S2.** Primers used for cloning of the 148 candidate FHals and the corresponding strains used for PCR gene amplification. **Table S3.** Kinetic parameters of FHals for which tryptophan is the metabolic substrate; comparison with the kinetic parameters of *Xszen*FHal. **Figure S1.** Matrice of the reference set. **Figure S2.** UHPLC traces of substrates and their corresponding chlorinated derivatives. **Figure S3.** MS spectra of 5-chlorotryptophan. **Figure S4.** Secondary metabolites from *Xenorhabdus szentirmaii.* **Figure S5.** A. UHPLC trace of the tryptophan bromination reaction by *Xszen*FHal. B. Time course of the conversion of tryptophan by *Xszen*FHal with NaBr over time. **Figure S6.** Plots for determination of kinetic parameters of *Xszen*FHal. A. Determination of the initial velocity. B. Michaelis-Menten kinetics. **Figure S7.** Percent identity matrix of the sequences of the 5-tryptophan halogenases.
