# Supplementary material for: XszenFHal, a novel tryptophan 5-halogenase from Xenorhabdus szentirmaii
Source: AMB Express. 2019 Oct 31;9:175. doi: 10.1186/s13568-019-0898-y (PMC6823310; doi:10.1186/s13568-019-0898-y)
Supplement: Supplementary file 1 — Additional file 1: Scheme S1. Synthesis of 5,7-dichloro-L-tryptophan (17). Table S1. Enzymes used for the reference set. Table S2. Primers used for cloning of the 148 candidate FHals and the corresponding strains used for PCR gene amplification. Table S3. Kinetic parameters of FHals for which tryptophan is the metabolic substrate; comparison with the kinetic parameters of XszenFHal. Figure S1. Matrice of the reference set. Figure S2. UHPLC traces of substrates and their corresponding chlorinated derivatives. Figure S3. MS spectra of 5-chlorotryptophan. Figure S4. Secondary metabolites from Xenorhabdus szentirmaii. Figure S5. A. UHPLC trace of the tryptophan bromination reaction by XszenFHal. B. Time course of the conversion of tryptophan by XszenFHal with NaBr over time. Figure S6. Plots for determination of kinetic parameters of XszenFHal. A. Determination of the initial velocity. B. Michaelis-Menten kinetics. Figure S7. Percent identity matrix of the sequences of the 5-tryptophan halogenases. [file 13568_2019_898_MOESM1_ESM.docx]

***Xszen*FHal, a novel tryptophan 5-halogenase from *Xenorhabdus szentirmaii***

Jérémy Domergue,^^[[1]](#footnote-1)^┴^ Diane Erdmann,^1┴^ Aurélie Fossey-Jouenne, Jean-Louis Petit,^1^ Adrien Debard,^1^ Véronique de Berardinis,^1^ Carine Vergne-Vaxelaire^1^ and Anne Zaparucha^1^*

Génomique métabolique, Genoscope, Institut François Jacob, CEA, CNRS, Univ Evry, Univ Paris-Saclay, 91057 Evry, France

┴ These authors have contributed equally to this work.

*Corresponding author:

Anne Zaparucha

anne.zaparucha@genoscope.cns.fr

0033 (0)1 60 87 45 78

Fax : 0033 (0)1 60 87 25 14

**Table of content**

**Schemes**

Scheme S1. Synthesis of 5,7-dichloro-L-tryptophan (**17**)

**Tables**

Table S1. Enzymes used for the reference set

Table S2. Primers used for cloning of the 148 candidate FHals and the corresponding strains used for PCR gene amplification

Table S3. Kinetic parameters of FHals for which tryptophan is the metabolic substrate; comparison with the kinetic parameters of *Xszen*FHal

**Figures**

Figure S1. Matrice of the reference set

Figure S2. UHPLC traces of substrates and their corresponding chlorinated derivatives

Figure S3. MS spectra of 5-chlorotryptophan

Figure S4. Secondary metabolites from *Xenorhabdus szentirmaii*

Figure S5. **A**. UHPLC trace of the tryptophan bromination reaction by *Xszen*FHal. **B**. Time course of the conversion of tryptophan by *Xszen*FHal with NaBr over time

Figure S6. Plots for determination of kinetic parameters of *Xszen*FHal. **A**. Determination of the initial velocity. **B**. Michaelis-Menten kinetics

Figure S7. Percent identity matrix of the sequences of the 5-tryptophan halogenases

**Schemes**

Scheme S1. Synthesis of 5,7-dichloro-L-tryptophan (**17**)([Heemstra and Walsh 2008](#_ENREF_1))

**Tables**

Table S1. Enzymes used for the reference set

|  | **Name** | **UniProtKB ID** | **Organisms** | **Substrates** | **Products** | **Reference** |
| --- | --- | --- | --- | --- | --- | --- |
| **1** | Putative halogenase | L8PEX5 | *Streptomyces viridochromogenes Tue57* | Phenol derivative | 2-Cl (avimamycin) | Hornung et al. ChemBioChem 2007, 8, 757 Weitnauer et al. Chem.Biol. 2001, 8, 569 |
| **2** | BhaA | O87676 | *Amycolatopsis balhimycina* | Phenol (Tyr) derivative | 2-Cl (balhimycin) | Hornung et al. ChemBioChem 2007, 8, 757 |
| **3** | Non heme halogenase | Q93N83 | *Streptomyces lavendulae* | Phenol (Tyr) or derivative/NRP | 2,6-Cl (complestatin) | Hornung et al. ChemBioChem 2007, 8, 757 Chiu et al. PNAS 2001, 98, 8548 |
| **4** | Halogenase | Q8KUG0 | *Actinosynnema pretiosum subsp. Auranticum* | Phenol/PKS | 2-Cl (ansamitocin) | Hornung et al. ChemBioChem 2007, 8, 757 Yu et al. PNAS, 2002, 99, 7968 |
| **5** | CalO3 | Q8KND5 | *Micromonospora echinospora (Micromonospora purpurea)* | Phenol/PKS | 2-I (calcheamicin) | Hornung et al. ChemBioChem 2007, 8, 757 |
| **6** | Putative halogenase | G9VYV6 | *Streptomyces antibioticus* | Phenol (Tyr) derivative | 2-Cl (simocyclinone) | Hornung et al. ChemBioChem 2007, 8, 757 |
| **7** | Trp-2- halogenase | Q0VZ69 | *Chondromyces crocatus* | Trp/PKS | 2-Cl (chondramin) | Rachid et al. Chem.Biol. 2006, 13, 667 |
| **8** | HrmQ | C1IHU5 | *Streptomyces griseoflavus W-384* | Pyrrole-ACP/NRP | 5-Cl (hormaomycin) | Heide at al. ChemBioChem 2008, 9, 1992 |
| **9** | PltA | Q4KCZ0 | *Pseudomonas fluorescens pf5* | Pyrrole-ACP/NRP | 4,5-Cl (pyoluteorin) | Dorrestein et al. PNAS 2005, 102, 13843 |
| **10** | PrnA | P95480 | *Pseudomonas fluorescens* | Trp | 7-Cl | Kirner et al. J. Bacteriol. 1998, 1939 |
| **11** | PrnC | P95482 | *Pseudomonas fluorescens* | Pyrrole derivative | 2-Cl | Kirner et al. J. Bacteriol. 1998, 1939 |
| **12** | RebH | Q8KHZ8 | *Lechevalieria aerocolonigenes (Nocardia aerocolonigenes) (Saccharothrix aerocolonigenes)* | Trp | 7-Cl | Yeh et al. PNAS 2005, 102, 3960 |
| **14** | PyrH | A4D0H5 | *Streptomyces rugosporus* | Trp | 5-Cl | Zehner et al. Chem.Biol. 2005, 12, 445 |
| **15** | KtzQ | A8CF75 | *Kutzneria sp. 744* | Trp | 7-Cl | Heemastra et al. JACS 2008, 130, 14024 |
| **16** | KtzR | A8CF74 | *Kutzneria sp. 744* | Trp | 6-Cl | Heemastra et al. JACS 2008, 130, 14024 |
| **17** | ThdH | A1E280 | *Streptomyces albogriseolus* | Trp | 6-Cl | Milbredt et al. ChemBioChem, 2014, 15, 1011 |
| **18** | HalB | Q71ME2 | *Actinoplanes sp. ATCC 33002* | n.d. | (pentachloro pseudilin) | Wynands et al. FEMS MicrobiolLett 2004, 237, 363 |
| **19** | CndH | B9ZUJ5 | *Chondromyces crocatus* | Phenol (Tyr)/PKS | 2-Cl (chondrochloren) | Buedenbender et al. JMolBiol 2009, 385, 520 |
| **20** | SgcC3 | Q8GMG6 | *Streptomyces globisporus* | Phenol (Tyr) derivative/NRP | 2-Cl (C-1027) | Lin et al. JACS 2007, 129, 12432 |
| **21** | CmlS | Q9AL91 | *Streptomyces venezuela* | AcetoacetylCoA | 3,3-diCl (chloramphenicol) | Podzeleinska et al. JMolBiol 2010, 397, 316 |

Table S2. Primers used for cloning of the 147 candidate F-Hals and the corresponding strains used for PCR gene amplification

| Hal ID | UniProtKB ID | Protein name | Organism | Strain used for gene PCR amplification | 5 PRIM primers* | 3 PRIM primers** |
| --- | --- | --- | --- | --- | --- | --- |
| FHal1 | A0A081KQX5 | 4-hydroxyphenylacetate 3-monooxygenase | *Lysinibacillus sphaericus* | DSMZ-1866 | AAAGAAGGAGATAGGATCATGCCAATAATGACAGGACAGC | GTGTAATGGATAGTGATCTTAATCATTTTTCAAAAAGTCTTC |
| FHal2 | A9L4W3 | Tryptophan halogenase | *Shewanella baltica (strain OS195)* | DSMZ-9439 | AAAGAAGGAGATAGGATCATGACAATAAAAACGATAGCGA | GTGTAATGGATAGTGATCTTATATGGATTGCAATCCGTA |
| FHal3 | C3BJM8 | 4-hydroxyphenylacetate-3-hydroxylase | *Bacillus pseudomycoides DSM 12442* | DSMZ-12442 | AAAGAAGGAGATAGGATCATGTATAGTAAACTTCAAAATGT | GTGTAATGGATAGTGATCTTACCTTAATGGATTTCCAGATAATTCCG |
| FHal4 | D7VWW6 | FAD dependent oxidoreductase | *Chryseobacterium gleum ATCC 35910* | DSMZ-16776 | AAAGAAGGAGATAGGATCATGAGCAAAGAATTTGTTGACGTTC | GTGTAATGGATAGTGATCTTATGCTTGATTTTGCTGTTCCTG |
| FHal5 | E6U0W0 | 4-hydroxyphenylacetate 3-monooxygenase, oxygenase subunit | *Bacillus cellulosilyticus (strain ATCC 21833 / DSM 2522 / FERM P-1141 / JCM 9156 / N-4)* | DSMZ-2522 | AAAGAAGGAGATAGGATCATGTCTATCATAACAGGACAACAATATATAAAGC | GTGTAATGGATAGTGATCTTATTGATTGTTCAAAAATGCTTGAATGT |
| FHal6 | F2IDP6 | FAD-dependent pyridine nucleotide-disulfide oxidoreductase | *Fluviicola taffensis (strain DSM 16823 / RW262 / RW262)* | DSMZ-16823 | AAAGAAGGAGATAGGATCATGACTAAAGAAGTAGATGTTGTTGTTATAGGTG | GTGTAATGGATAGTGATCTTACATTTTTACGATGTGCGCTACC |
| FHal7 | Q47XR9 | Putative tryptophan halogenase | *Colwellia psychrerythraea (strain 34H / ATCC BAA-681)* | ATCC-BAA-681D5 | AAAGAAGGAGATAGGATCATGAATAATCTATCAGAAGTAAA | GTGTAATGGATAGTGATCTTAGGCTTTAGAATTACAATATT |
| FHal8 | Q47XV2 | Putative tryptophan halogenase | *Colwellia psychrerythraea (strain 34H / ATCC BAA-681)* | ATCC-BAA-681D5 | AAAGAAGGAGATAGGATCATGAAAGATAAAATTGAAAATATTG | GTGTAATGGATAGTGATCTTAATAGTCTGATTTACAATAAC |
| FHal9 | Q47XV3 | Putative tryptophan halogenase | *Colwellia psychrerythraea (strain 34H / ATCC BAA-681)* | ATCC-BAA-681D5 | AAAGAAGGAGATAGGATCATGGGCAAAGCTATTAAAAAAA | GTGTAATGGATAGTGATCTTACTTCTCTTTTAACTTATTGAGTAACTCTC |
| FHal10 | Q487J3 | Putative tryptophan halogenase | *Colwellia psychrerythraea (strain 34H / ATCC BAA-681)* | ATCC-BAA-681D5 | AAAGAAGGAGATAGGATCATGAATGAACAAGTGAAACAAGT | GTGTAATGGATAGTGATCTTATATTTTCTGAAAACCATATTG |
| FHal11 | Q487J4 | Putative tryptophan halogenase | *Colwellia psychrerythraea (strain 34H / ATCC BAA-681)* | ATCC-BAA-681D5 | AAAGAAGGAGATAGGATCATGATGGCCTTAGATAATAATC | GTGTAATGGATAGTGATCTTATACTTGAGTAAACCCATATTTAATTACTTTTTC |
| FHal12 | Q487M4 | Putative tryptophan halogenase | *Colwellia psychrerythraea (strain 34H / ATCC BAA-681)* | ATCC-BAA-681D5 | AAAGAAGGAGATAGGATCATGAATAGTCCAGTTAAAAAAG | GTGTAATGGATAGTGATCTTACTGCTGACAGAATATTCT |
| FHal13 | Q4J6K2 | 4-hydroxyphenylacetate 3-monooxygenase, oxygenase component | *Sulfolobus acidocaldarius (strain ATCC 33909 / DSM 639 / JCM 8929 / NBRC 15157 / NCIMB 11770)* | DSMZ-639 | AAAGAAGGAGATAGGATCATGATTAGAAGGGGAGAAGAATATATTAAAAG | GTGTAATGGATAGTGATCTTACGCCTTAGGATTAGGCATCTG |
| FHal14 | Q5E6L0 | Dehydrogenase | *Vibrio fischeri (strain ATCC 700601 / ES114)* | DSMZ-507 | AAAGAAGGAGATAGGATCATGTTATCACCACAAACAACCCAAG | GTGTAATGGATAGTGATCTTATGGCTTACATAATTCAGCAAC |
| FHal15 | Q8EMH2 | 4-hydroxyphenylacetate-3-hydroxylase | *Oceanobacillus iheyensis (strain DSM 14371 / JCM 11309 / KCTC 3954 / HTE831)* | DSMZ-14371 | AAAGAAGGAGATAGGATCATGCCAGCTAAAACAGGACAGC | GTGTAATGGATAGTGATCTTACTTTAAGACACTTCCTGTTGGAACG |
| FHal16 | W1J423 | Tryptophan 5-halogenase | *Xenorhabdus szentirmaii DSM 16338* | DSM-16338 | AAAGAAGGAGATAGGATCATGATAAATTCAGTATTGATAGT | GTGTAATGGATAGTGATCTTAATTCTTGTTTTTTAAATCATG |
| FHal17 | W4R199 | 4-hydroxyphenylacetate 3-monooxygenase | *Bacillus akibai (strain ATCC 43226 / DSM 21942 / JCM 9157 / 1139)* | DSM-21942 | AAAGAAGGAGATAGGATCATGATAACAACAGGTAAAGATTATATTG | GTGTAATGGATAGTGATCTTATTTATCTTGCAAAAATGTTTCAATTTGT |
| FHal18 | A3QBY6 | Tryptophan halogenase | *Shewanella loihica (strain ATCC BAA-1088 / PV-4)* | DSMZ-17748 | AAAGAAGGAGATAGGATCATGGACATTAAGCGTATCCTTATATTGGGC | GTGTAATGGATAGTGATCTTAACATTTTTGTAAGCCATATACCGCGA |
| FHal19 | A3QIT9 | Tryptophan halogenase | *Shewanella loihica (strain ATCC BAA-1088 / PV-4)* | DSMZ-17748 | AAAGAAGGAGATAGGATCATGACCCCTTTCGCGCAAAC | GTGTAATGGATAGTGATCTTAACGGCATAGCTCGACCACC |
| FHal20 | A3ZZ99 | Probable alkylhalidase | *Blastopirellula marina DSM 3645* | DSMZ-3645 | AAAGAAGGAGATAGGATCATGATCGCCAGCGAGAAACCG | GTGTAATGGATAGTGATCTTAGTCCTGCTCTTTGGTTGACATC |
| FHal21 | A4YFG2 | 4-hydroxyphenylacetate 3-monooxygenase oxygenase component | *Metallosphaera sedula (strain ATCC 51363 / DSM 5348)* | DSMZ-5348 | AAAGAAGGAGATAGGATCATGATAAGGAAAGGGAGTGACTACATAGAG | GTGTAATGGATAGTGATCTTAGGCCTTTGGATTGGGCATC |
| FHal22 | A6BZI3 | Probable alkylhalidase | *Planctomyces maris DSM 8797* | DSMZ-8797 | AAAGAAGGAGATAGGATCATGCTTGATACGGACCTGAAAGAACAG | GTGTAATGGATAGTGATCTTATTTTTTCATCGTAACAGGGG |
| FHal23 | A6WL61 | Tryptophan halogenase | *Shewanella baltica (strain OS185)* | DSMZ-9439 | AAAGAAGGAGATAGGATCATGAAAGTGAAACAAGCAATCAGAAAAATC | GTGTAATGGATAGTGATCTTACGAATGCTTAGGCTGAAAATACTGC |
| FHal24 | A8FSJ7 | Tryptophan halogenase | *Shewanella sediminis (strain HAW-EB3)* | DSMZ-17055 | AAAGAAGGAGATAGGATCATGGATATTAAGAGAGTTGTAATTGTTGG | GTGTAATGGATAGTGATCTTATACCTCTTGCAAGCCATAGCG |
| FHal25 | A9W9T9 | 4-hydroxyphenylacetate 3-hydroxylase | *Chloroflexus aurantiacus (strain ATCC 29366 / DSM 635 / J-10-fl)* | DSMZ-635 | AAAGAAGGAGATAGGATCATGACGGTATCCGAAGCGATGAC | GTGTAATGGATAGTGATCTTAATGCTGACCCATCACAACATTGAC |
| FHal26 | B1KEB2 | Tryptophan halogenase | *Shewanella woodyi (strain ATCC 51908 / MS32)* | DSMZ-12036 | AAAGAAGGAGATAGGATCATGCACTCACCCATAAAAAAGA | GTGTAATGGATAGTGATCTTACTTTAAGCCGCAATATTGAG |
| FHal27 | B1KET1 | Tryptophan halogenase | *Shewanella woodyi (strain ATCC 51908 / MS32)* | DSMZ-12036 | AAAGAAGGAGATAGGATCATGGATAAACCAACACAAAAAATC | GTGTAATGGATAGTGATCTTAAATTTGGCTAAAGCCATATTG |
| FHal28 | B1KFL8 | Tryptophan halogenase | *Shewanella woodyi (strain ATCC 51908 / MS32)* | DSMZ-12036 | AAAGAAGGAGATAGGATCATGACGAAACCAATCACACAGG | GTGTAATGGATAGTGATCTTATTGAGACACTGCTTTGCAG |
| FHal29 | B1KFL9 | Tryptophan halogenase | *Shewanella woodyi (strain ATCC 51908 / MS32)* | DSMZ-12036 | AAAGAAGGAGATAGGATCATGACCCCAGAATCTATTAAAAA | GTGTAATGGATAGTGATCTTAAATTTTAGGAGCTGGGCAA |
| FHal30 | B1KFM0 | Tryptophan halogenase | *Shewanella woodyi (strain ATCC 51908 / MS32)* | DSMZ-12036 | AAAGAAGGAGATAGGATCATGATGGAAAATTCAAAACAGGTGATAAGG | GTGTAATGGATAGTGATCTTACGCCTCATCTGCTGCAACTT |
| FHal31 | B1KHC5 | Tryptophan halogenase | *Shewanella woodyi (strain ATCC 51908 / MS32)* | DSMZ-12036 | AAAGAAGGAGATAGGATCATGGATAATAAAATCAAAAAAGTCG | GTGTAATGGATAGTGATCTTAGACTACCGTTGATTTACAG |
| FHal32 | B1KHC8 | Tryptophan halogenase | *Shewanella woodyi (strain ATCC 51908 / MS32)* | DSMZ-12036 | AAAGAAGGAGATAGGATCATGCACCAAGCGATCAGTAATATCAT | GTGTAATGGATAGTGATCTTATAAAGTAGGGAAACCAAACT |
| FHal33 | B1KIV0 | Tryptophan halogenase | *Shewanella woodyi (strain ATCC 51908 / MS32)* | DSMZ-12036 | AAAGAAGGAGATAGGATCATGAAGATTAAACGAATTGCCATTGTTGGT | GTGTAATGGATAGTGATCTTAGCCATTAGGGCTCATAGCTGCTT |
| FHal34 | B1KLX6 | Tryptophan halogenase | *Shewanella woodyi (strain ATCC 51908 / MS32)* | DSMZ-12036 | AAAGAAGGAGATAGGATCATGCAAACAGCCTCAGCAACACAG | GTGTAATGGATAGTGATCTTATTTACATAGCTCTACTATCAG |
| FHal35 | B7J6K4 | Non-heme halogenase, putative | *Acidithiobacillus ferrooxidans (strain ATCC 23270 / DSM 14882 / NCIB 8455)* | DSMZ-14882 | AAAGAAGGAGATAGGATCATGGGAAAAAATGAAATGTCCAC | GTGTAATGGATAGTGATCTTATACTCGTATGTCTTCTTTACGCCGC |
| FHal36 | B8E576 | Tryptophan halogenase | *Shewanella baltica (strain OS223)* | DSMZ-9439 | AAAGAAGGAGATAGGATCATGAATAAAATCAAAGATATCGTC | GTGTAATGGATAGTGATCTTATTTATGTGTCGATAAGTGCGCTA |
| FHal37 | C5BK75 | Tryptophan halogenase | *Teredinibacter turnerae (strain ATCC 39867 / T7901)* | ATCC-39867 | AAAGAAGGAGATAGGATCATGAGCAAGAGCGTCTTGATTGTTG | GTGTAATGGATAGTGATCTTATGCACGAGCCTTGGCTTTGG |
| FHal38 | C5BK76 | Tryptophan halogenase | *Teredinibacter turnerae (strain ATCC 39867 / T7901)* | ATCC-39867 | AAAGAAGGAGATAGGATCATGAACCCCTCGCAAGTGAAGC | GTGTAATGGATAGTGATCTTAGACTCTTGAAGATTCGGC |
| FHal39 | C5BN16 | Tryptophan halogenase PrnA | *Teredinibacter turnerae (strain ATCC 39867 / T7901)* | ATCC-39867 | AAAGAAGGAGATAGGATCATGAGCGAACAAAAAACTAAGG | GTGTAATGGATAGTGATCTTATATTGCACAATACTCCGCCAGG |
| FHal40 | C6XIP4 | Monooxygenase FAD-binding | *Hirschia baltica (strain ATCC 49814 / DSM 5838 / IFAM 1418)* | DSMZ-5838 | AAAGAAGGAGATAGGATCATGCAAAATGAAGTCTGTGACGTTGCC | GTGTAATGGATAGTGATCTTAAGCGTCTTCTTTTGTCAGCGCATC |
| FHal41 | C6XNI6 | Tryptophan halogenase | *Hirschia baltica (strain ATCC 49814 / DSM 5838 / IFAM 1418)* | DSMZ-5838 | AAAGAAGGAGATAGGATCATGAACCAAGCTGCGATTTC | GTGTAATGGATAGTGATCTTAGTATAATTGTTTCAAAACTTCT |
| FHal42 | C6XNI7 | Tryptophan halogenase | *Hirschia baltica (strain ATCC 49814 / DSM 5838 / IFAM 1418)* | DSMZ-5838 | AAAGAAGGAGATAGGATCATGACTGATAGAAATATTCGTAAA | GTGTAATGGATAGTGATCTTACCGACCCTCCCCCAAGTC |
| FHal43 | C6XNI9 | Tryptophan halogenase | *Hirschia baltica (strain ATCC 49814 / DSM 5838 / IFAM 1418)* | DSMZ-5838 | AAAGAAGGAGATAGGATCATGGTAGATAATAGAGTGAAATC | GTGTAATGGATAGTGATCTTAATTCAAGCTCTCCTTTGGA |
| FHal44 | C6XS27 | Tryptophan halogenase | *Hirschia baltica (strain ATCC 49814 / DSM 5838 / IFAM 1418)* | DSMZ-5838 | AAAGAAGGAGATAGGATCATGAACCAAGACAATAATTCTCATCA | GTGTAATGGATAGTGATCTTAGTAAAAATCTAGATTAGCCTG |
| FHal45 | C7M500 | Tryptophan halogenase | *Capnocytophaga ochracea (strain ATCC 27872 / DSM 7271 / JCM 12966 / VPI 2845)* | DSMZ-7271 | AAAGAAGGAGATAGGATCATGAAAAAAGAAGTAACAGATGTA | GTGTAATGGATAGTGATCTTATGCCTCCTTCATTCCCAAACC |
| FHal46 | C7PAY7 | Tryptophan halogenase | *Chitinophaga pinensis (strain ATCC 43595 / DSM 2588 / NCIB 11800 / UQM 2034)* | DSMZ-2588 | AAAGAAGGAGATAGGATCATGACTACAGAAAAGGTAGACG | GTGTAATGGATAGTGATCTTACTTGTCCTGTTTTAAGATTTC |
| FHal47 | C7R9X3 | Tryptophan halogenase | *Kangiella koreensis (strain DSM 16069 / KCTC 12182 / SW-125)* | DSMZ-16069 | AAAGAAGGAGATAGGATCATGCCTGAGTTAAACTCGCAA | GTGTAATGGATAGTGATCTTACGATGTTGCGCATAATTGC |
| FHal48 | C7RB81 | Tryptophan halogenase | *Kangiella koreensis (strain DSM 16069 / KCTC 12182 / SW-125)* | DSMZ-16069 | AAAGAAGGAGATAGGATCATGATAAAAAAAGTATTGATTGTTG | GTGTAATGGATAGTGATCTTATACCCAACCATCGGGAAAGC |
| FHal49 | D5WPM2 | 4-hydroxyphenylacetate 3-monooxygenase, oxygenase subunit | *Kyrpidia tusciae (strain DSM 2912 / NBRC 15312 / T2)* | DSMZ-2912 | AAAGAAGGAGATAGGATCATGGGGATCCGAACAGGGC | GTGTAATGGATAGTGATCTTAGTCGTCATTGCGATACAAAAACTGC |
| FHal50 | E4RWY0 | Monooxygenase FAD-binding protein | *Leadbetterella byssophila (strain DSM 17132 / KACC 11308 / 4M15)* | DSMZ-17132 | AAAGAAGGAGATAGGATCATGAAAAAGGAAGCGGTAGATGTGTTAGTC | GTGTAATGGATAGTGATCTTAATATTCCTCCGGACTACCCCCG |
| FHal51 | E8RV31 | Tryptophan halogenase | *Asticcacaulis excentricus (strain ATCC 15261 / DSM 4724 / VKM B-1370 / CB 48)* | DSM-4724 | AAAGAAGGAGATAGGATCATGAACGTCAAAGGCATGTAC | GTGTAATGGATAGTGATCTTACGCGCTGACGAAGTGAGG |
| FHal52 | F2GAR4 | Tryptophan halogenase | *Alteromonas macleodii (strain DSM 17117 / Deep ecotype)* | DSMZ-17117 | AAAGAAGGAGATAGGATCATGACAACAGCAACTCCCATAACGC | GTGTAATGGATAGTGATCTTAAACGGCCTGCAGGCCG |
| FHal53 | F2GC26 | Tryptophan halogenase | *Alteromonas macleodii (strain DSM 17117 / Deep ecotype)* | DSMZ-17117 | AAAGAAGGAGATAGGATCATGCAAGATCCTCATAATAAACCG | GTGTAATGGATAGTGATCTTAACTCATTGCGCGCTTATACTTTGC |
| FHal54 | G3IYZ1 | Monooxygenase, FAD-binding protein | *Methylobacter tundripaludum SV96* | ATCC-BAA-1195 | AAAGAAGGAGATAGGATCATGACAATTAAGAATAATAAAGGCGAAA | GTGTAATGGATAGTGATCTTATCGGCATAACTCAACCAAAGTATCC |
| FHal55 | G4SW40 | Monooxygenase FAD-binding | *Methylomicrobium alcaliphilum (strain DSM 19304 / NCIMB 14124 / VKM B-2133 / 20Z)* | DSMZ-19304 | AAAGAAGGAGATAGGATCATGAGAGCTGAAAAAGGCAATATC | GTGTAATGGATAGTGATCTTAAGGCTCGCGACACAATTCG |
| FHal56 | G6DXN6 | Tryptophan halogenase | *Shewanella baltica OS625* | DSMZ-9439 | AAAGAAGGAGATAGGATCATGCAGCAAGCAACTCACACAGC | GTGTAATGGATAGTGATCTTAAATCTTAGGGAAACCAAATTGCCTG |
| FHal57 | G8R624 | Flavin-dependent dehydrogenase | *Owenweeksia hongkongensis (strain DSM 17368 / JCM 12287 / NRRL B-23963)* | DSMZ-17368 | AAAGAAGGAGATAGGATCATGAGAAAATGTGATGTAGTAGT | GTGTAATGGATAGTGATCTTACTCTTCAGGCTTATCCATTTGGATGTC |
| FHal58 | G8TSH0 | 4-hydroxyphenylacetate 3-monooxygenase, oxygenase subunit | *Sulfobacillus acidophilus (strain ATCC 700253 / DSM 10332 / NAL)* | DSMZ-10332 | AAAGAAGGAGATAGGATCATGGGGATTCGGACCGGC | GTGTAATGGATAGTGATCTTACGACCGCGCCTCCTTTG |
| FHal59 | H1YRA5 | Monooxygenase FAD-binding | *Shewanella baltica OS183* | DSMZ-9439 | AAAGAAGGAGATAGGATCATGTCTACACAGGTTCACGATTTATCAGC | GTGTAATGGATAGTGATCTTAGACCTCTGCGAATGCTGGAG |
| FHal60 | H2BV11 | Monooxygenase FAD-binding | *Gillisia limnaea DSM 15749* | DSMZ-15749 | AAAGAAGGAGATAGGATCATGAAAAAGGAAATTGCAGATGT | GTGTAATGGATAGTGATCTTAAAGCTGATTTTCTTCCATTTCAATAAG |
| FHal61 | I0JKQ9 | 4-hydroxyphenylacetate-3-hydroxylase | *Halobacillus halophilus (strain ATCC 35676 / DSM 2266 / JCM 20832 / NBRC 102448/ NCIMB 2269)* | DSMZ-2266 | AAAGAAGGAGATAGGATCATGCCAGCGAAAACAGGTGC | GTGTAATGGATAGTGATCTTATACGTTTGTATAGTTTGGTTTGGACG |
| FHal62 | I0JQ92 | 4-hydroxyphenylacetate-3-hydroxylase | *Halobacillus halophilus (strain ATCC 35676 / DSM 2266 / JCM 20832 / NBRC 102448/ NCIMB 2269)* | DSMZ-2266 | AAAGAAGGAGATAGGATCATGCCTGCTATATCAGGTGAT | GTGTAATGGATAGTGATCTTAGCTTTCTTTTAGAAACCATGAAACT |
| FHal63 | I7DTD4 | Putative tryptophan halogenase | *Phaeobacter inhibens (strain ATCC 700781 / DSM 17395 / CIP 105210 / NBRC 16654 / BS107)* | DSMZ-17395 | AAAGAAGGAGATAGGATCATGAACAATCGCTTAAGGAAGATCACC | GTGTAATGGATAGTGATCTTAGAACAATTGCAGGTCTTTCTGACCG |
| FHal64 | K4KGG8 | Tryptophan halogenase | *Simiduia agarivorans (strain DSM 21679 / JCM 13881 / BCRC 17597 / SA1)* | DSMZ-21679 | AAAGAAGGAGATAGGATCATGACAAAACGCATCATCATAGTTGG | GTGTAATGGATAGTGATCTTAATATTGACCTTTCGCGTGC |
| FHal65 | K4KJJ4 | Tryptophan halogenase | *Simiduia agarivorans (strain DSM 21679 / JCM 13881 / BCRC 17597 / SA1)* | DSMZ-21679 | AAAGAAGGAGATAGGATCATGCCGGTGAAAACCAAGGTATC | GTGTAATGGATAGTGATCTTATCTGCCACAGAAAGACTTCAGA |
| FHal66 | K4KM68 | Tryptophan halogenase | *Simiduia agarivorans (strain DSM 21679 / JCM 13881 / BCRC 17597 / SA1)* | DSMZ-21679 | AAAGAAGGAGATAGGATCATGCAACCAAAACGACTGGT | GTGTAATGGATAGTGATCTTATTTTTTATGGAGTAATTGCAG |
| FHal67 | K4KQI7 | Tryptophan halogenase | *Simiduia agarivorans (strain DSM 21679 / JCM 13881 / BCRC 17597 / SA1)* | DSMZ-21679 | AAAGAAGGAGATAGGATCATGGAAAACAAAGCGGTAAGAAAAGTAGTG | GTGTAATGGATAGTGATCTTAAACGGCTTGCAGGCCATAGTG |
| FHal68 | K4KR94 | Tryptophan halogenase | *Simiduia agarivorans (strain DSM 21679 / JCM 13881 / BCRC 17597 / SA1)* | DSMZ-21679 | AAAGAAGGAGATAGGATCATGAAAAAACCGATTCGAAAAGT | GTGTAATGGATAGTGATCTTACATCGGCGGCTTGGC |
| FHal69 | K6ZEX7 | FADH2 O2-dependent halogenase I | *Glaciecola pallidula DSM 14239 = ACAM 615* | DSMZ-14239 | AAAGAAGGAGATAGGATCATGAAAAAACAACAGAGAAAAATTA | GTGTAATGGATAGTGATCTTAAGTGTTAACGCTCGATGGG |
| FHal70 | K6ZH11 | Tryptophan halogenase | *Glaciecola pallidula DSM 14239 = ACAM 615* | DSMZ-14239 | AAAGAAGGAGATAGGATCATGAAAAAACTGCTGATTTTAGG | GTGTAATGGATAGTGATCTTAGATTAATCCTAAACTTGCGT |
| FHal71 | K7A056 | Tryptophan halogenase | *Glaciecola pallidula DSM 14239 = ACAM 615* | DSMZ-14239 | AAAGAAGGAGATAGGATCATGCACAAGCCCTTAAGAAAAATAG | GTGTAATGGATAGTGATCTTAAATTTTTGGTAAGCCATATAGTTTGAT |
| FHal72 | K7A5S3 | Tryptophan halogenase | *Glaciecola pallidula DSM 14239 = ACAM 615* | DSMZ-14239 | AAAGAAGGAGATAGGATCATGTCTGACGCTATAAAAACGATTG | GTGTAATGGATAGTGATCTTACTTCATTTGTGTGTTTGCAA |
| FHal73 | N8QII6 | Uncharacterized protein | *Acinetobacter johnsonii CIP 64.6* | DSMZ-6963 | AAAGAAGGAGATAGGATCATGAGCACCATGCAACAGACAGATG | GTGTAATGGATAGTGATCTTAAGTTTCATCGGTCAGCGACTCTTTTTG |
| FHal74 | N8ZV53 | Uncharacterized protein | *Acinetobacter baylyi DSM 14961 = CIP 107474* | INTERNE | AAAGAAGGAGATAGGATCATGCAACAGATTCAAGACAATAACTTAC | GTGTAATGGATAGTGATCTTAAACATCCAGTACTTTTGCTTGGGG |
| FHal75 | Q11R94 | Flavoprotein/dehydrogenase | *Cytophaga hutchinsonii (strain ATCC 33406 / NCIMB 9469)* | DSMZ-1761 | AAAGAAGGAGATAGGATCATGCAACGGGAAAAAGTTGATGTG | GTGTAATGGATAGTGATCTTATGCCTCTTCACCCATCTGAATG |
| FHal76 | Q12KP0 | Tryptophan halogenase | *Shewanella denitrificans (strain OS217 / ATCC BAA-1090 / DSM 15013)* | DSMZ-15013 | AAAGAAGGAGATAGGATCATGAAAATTAAGCGAATTGCGATTGTTG | GTGTAATGGATAGTGATCTTATGCCCTAAGCGCTTCAGTAGG |
| FHal77 | Q12ML3 | Tryptophan halogenase | *Shewanella denitrificans (strain OS217 / ATCC BAA-1090 / DSM 15013)* | DSMZ-15013 | AAAGAAGGAGATAGGATCATGAAGTCAGAAATTTCAAAAGTGGT | GTGTAATGGATAGTGATCTTATCGCAATAATAAACTGGCAAGGAAG |
| FHal78 | Q21GU4 | Tryptophan halogenase | *Saccharophagus degradans (strain 2-40 / ATCC 43961 / DSM 17024)* | DSMZ-17024 | AAAGAAGGAGATAGGATCATGAAACAAAATAAAATTAAAAAAGTGG | GTGTAATGGATAGTGATCTTAGATAGGTATGGGTGGCGCTTTG |
| FHal79 | Q21GW5 | Tryptophan halogenase | *Saccharophagus degradans (strain 2-40 / ATCC 43961 / DSM 17024)* | DSMZ-17024 | AAAGAAGGAGATAGGATCATGAAGCCACTGCAAAAAATAG | GTGTAATGGATAGTGATCTTATTTGCTGCTGCTCGCAC |
| FHal80 | Q21HS1 | Tryptophan halogenase | *Saccharophagus degradans (strain 2-40 / ATCC 43961 / DSM 17024)* | DSMZ-17024 | AAAGAAGGAGATAGGATCATGCACAGTAAGCAAGTTAAGAAGATAGT | GTGTAATGGATAGTGATCTTAGAATTTGCCGTCGGCGAGTGTAG |
| FHal81 | Q21KW6 | Tryptophan halogenase | *Saccharophagus degradans (strain 2-40 / ATCC 43961 / DSM 17024)* | DSMZ-17024 | AAAGAAGGAGATAGGATCATGAATAACAAAATAAAAAACGTAG | GTGTAATGGATAGTGATCTTACTCTTGAGCGAACGCAGCAC |
| FHal82 | Q21KW9 | Tryptophan halogenase | *Saccharophagus degradans (strain 2-40 / ATCC 43961 / DSM 17024)* | DSMZ-17024 | AAAGAAGGAGATAGGATCATGAGAGAGCTTACTAAGCGC | GTGTAATGGATAGTGATCTTATTGTTTTTTTGGCAAACCTAC |
| FHal83 | Q21LR2 | Tryptophan halogenase | *Saccharophagus degradans (strain 2-40 / ATCC 43961 / DSM 17024)* | DSMZ-17024 | AAAGAAGGAGATAGGATCATGATCAAAAAAATAGTGGTGTTA | GTGTAATGGATAGTGATCTTAAAGCTTTTGCATACCGAAC |
| FHal84 | Q5QXF8 | FAD-binding protein | *Idiomarina loihiensis (strain ATCC BAA-735 / DSM 15497 / L2-TR)* | DSMZ-15497 | AAAGAAGGAGATAGGATCATGGAAGAGTCGATTTATGATGTGGTG | GTGTAATGGATAGTGATCTTATCCACATAATTCTGCCAATACG |
| FHal85 | Q6MN71 | Tryptophan halogenase | *Bdellovibrio bacteriovorus (strain ATCC 15356 / DSM 50701 / NCIB 9529 / HD100)* | DSMZ-50701 | AAAGAAGGAGATAGGATCATGAGCACACTTTCAGAACATATCG | GTGTAATGGATAGTGATCTTAGACGTCGGCTTCCATGATCC |
| FHal86 | Q7N9R8 | Complete genome; segment 1/17 | *Photorhabdus luminescens subsp. laumondii (strain TT01)* | DSMZ-15139 | AAAGAAGGAGATAGGATCATGTCAGAAAACATGAAACAAAAATCGTC | GTGTAATGGATAGTGATCTTAGCGGTTGGAAGGCCAAATAAAATC |
| FHal87 | Q7UYT6 | Probable alkylhalidase | *Rhodopirellula baltica (strain SH1)* | DSMZ-10527 | AAAGAAGGAGATAGGATCATGTCTGCTCAGCAACCTTTCGG | GTGTAATGGATAGTGATCTTAAGTCGACATGGGTTTGGTTTCG |
| FHal88 | Q9K693 | 4-hydroxyphenylacetate-3-hydroxylase | *Bacillus halodurans (strain ATCC BAA-125 / DSM 18197 / FERM 7344 / JCM 9153 / C-125)* | DSMZ-497 | AAAGAAGGAGATAGGATCATGGCGATCATTAAGGGAAGTG | GTGTAATGGATAGTGATCTTAACGTTTTTTTAAAAACGATTGC |
| FHal89 | V4NC16 | Uncharacterized protein | *Asticcacaulis benevestitus DSM 16100 = ATCC BAA-896* | DSMZ-16100 | AAAGAAGGAGATAGGATCATGCTGGCGAAGACGCTGG | GTGTAATGGATAGTGATCTTACGAGGTTCGCGAGGCG |
| FHal90 | V4PQ38 | Uncharacterized protein | *Asticcacaulis benevestitus DSM 16100 = ATCC BAA-896* | DSMZ-16100 | AAAGAAGGAGATAGGATCATGAGCGCCATAAAAAAAATCG | GTGTAATGGATAGTGATCTTAGGCCTTGCAGCCGGC |
| FHal91 | V4PWR0 | Uncharacterized protein | *Asticcacaulis benevestitus DSM 16100 = ATCC BAA-896* | DSMZ-16100 | AAAGAAGGAGATAGGATCATGTCGCAAGGCACCATC | GTGTAATGGATAGTGATCTTAGGCGCTTTCAGATAGGCGG |
| FHal92 | V4PZN0 | Uncharacterized protein | *Asticcacaulis benevestitus DSM 16100 = ATCC BAA-896* | DSMZ-16100 | AAAGAAGGAGATAGGATCATGACCAATCCAGCTATCAGCAGC | GTGTAATGGATAGTGATCTTATAGACTGGCGGCCTTGCAATAG |
| FHal93 | V6IXL8 | MFS transporter | *Sporolactobacillus laevolacticus DSM 442* | DSMZ-442 | AAAGAAGGAGATAGGATCATGTCATGTATCAACGGCAAAA | GTGTAATGGATAGTGATCTTACTTTTGCCGATTAAGAAATTC |
| FHal94 | W0F275 | Tryptophan halogenase | *Niabella soli DSM 19437* | DSMZ-19437 | AAAGAAGGAGATAGGATCATGCAAACTGAAAAAACAGATGT | GTGTAATGGATAGTGATCTTATCCCTTTTCTTTAAACCCGTTA |
| FHal95 | W1J6Q1 | Putative 4-hydroxyphenylacetate 3-monooxygenase,oxygenase component (4-HPA 3-monooxygenase large component) (4-HPA 3-hydroxylase) | *Xenorhabdus szentirmaii DSM 16338* | DSM-16338 | AAAGAAGGAGATAGGATCATGAAAAATATGATGAGAACTGGTGAAG | GTGTAATGGATAGTGATCTTAGAGCATCTTAATTTTTTCAAC |
| FHal96 | W1J792 | Putative 4-hydroxyphenylacetate 3-monooxygenase,oxygenase component (4-HPA 3-monooxygenase large component) (4-HPA 3-hydroxylase) | *Xenorhabdus szentirmaii DSM 16338* | DSM-16338 | AAAGAAGGAGATAGGATCATGATAAGAACCGGAGAAAGATATTTGG | GTGTAATGGATAGTGATCTTAGATTGTTTTTATCTTTTCGACCAACGC |
| FHal97 | A0A077BUF9 | FAD binding domain protein | *Stenotrophomonas maltophilia* | DSM-50170 | AAAGAAGGAGATAGGATCATGAGTACTGCTGTGGATGCTGTCGAACG | GTGTAATGGATAGTGATCTTAGGCGCTGCACAATTCCTCCAG |
| FHal98 | A0A077SZB4 | 4-hydroxyphenylacetate 3-monooxygenase large chain | *Pseudomonas aeruginosa* | DSMZ-22644 | AAAGAAGGAGATAGGATCATGAAACCCGAAGATTTCCGTGCCTCTGC | GTGTAATGGATAGTGATCTTATTGGCGGATGCGATCGAGCACGTTG |
| FHal99 | A1R2G2 | Putative 4-hydroxyphenylacetate 3-monooxygenase | *Arthrobacter aurescens (strain TC1)* | ATCC-BAA-1386 | AAAGAAGGAGATAGGATCATGAGGACCGGAAAAGAGTACCTG | GTGTAATGGATAGTGATCTTAGATCCCTTCCGATTCGGTGGC |
| FHal100 | A3K7D3 | Putative phenol hydroxylase large subunit | *Sagittula stellata E-37* | DSMZ-11524 | AAAGAAGGAGATAGGATCATGAAAGACCAAACGACACAAGGCACCGC | GTGTAATGGATAGTGATCTTACTGGAAGCCGCCCTTGAGGC |
| FHal101 | A4FJG0 | Tryptophan halogenase | *Saccharopolyspora erythraea (strain NRRL 23338)* | DSMZ-40517 | AAAGAAGGAGATAGGATCATGGAACACAAGGTGCAGAAGGTCGTGA | GTGTAATGGATAGTGATCTTACCGCGCGGAGACAGTCGGATGAATG |
| FHal102 | B2T674 | Tryptophan halogenase | *Burkholderia phytofirmans (strain DSM 17436 / PsJN)* | DSMZ-17436 | AAAGAAGGAGATAGGATCATGAGTACACATTCGTCAAAGCGCACGAC | GTGTAATGGATAGTGATCTTACCGCTGCATGCACACCTCATG |
| FHal103 | B9L2J3 | 4-hydroxyphenylacetate 3-monooxygenase, oxygenase component | *Thermomicrobium roseum (strain ATCC 27502 / DSM 5159 / P-2)* | DSMZ-5159 | AAAGAAGGAGATAGGATCATGGCACAAGTCATCCAGACGTCGCCC | GTGTAATGGATAGTGATCTTACCGTTCGCGACCGTTCGTTTCCC |
| FHal104 | B9L565 | 4-hydroxyphenylacetate 3-monooxygenase oxygenase component (4-HPA 3-monooxygenase large component) (4-HPA 3-hydroxylase) | *Thermomicrobium roseum (strain ATCC 27502 / DSM 5159 / P-2)* | DSMZ-5159 | AAAGAAGGAGATAGGATCATGACGCAACCACTCACCGGCACAGAG | GTGTAATGGATAGTGATCTTACACCGATTCCGCTGCTCGTTGCTG |
| FHal105 | B9L567 | Pyoverdin chromophore biosynthetic protein PvcC | *Thermomicrobium roseum (strain ATCC 27502 / DSM 5159 / P-2)* | DSMZ-5159 | AAAGAAGGAGATAGGATCATGGGAGCACGGACGGGCAAGG | GTGTAATGGATAGTGATCTTATGCAACTCCCCCACGCAGCT |
| FHal106 | C4RL56 | Alkylhalidase cmlS | *Micromonospora sp. ATCC 39149* | ATCC-39149 | AAAGAAGGAGATAGGATCATGCGTGGACATCAAACCGAAGGGCG | GTGTAATGGATAGTGATCTTACACCTCGTATTCGACGCCGACCCCGC |
| FHal107 | C7PXC2 | Tryptophan halogenase | *Catenulispora acidiphila (strain DSM 44928 / NRRL B-24433 / NBRC 102108 / JCM 14897)* | DSMZ-44928 | AAAGAAGGAGATAGGATCATGACTGAGCAGAACACAGTGAAAAC | GTGTAATGGATAGTGATCTTACAACCCGGCCCTCGCTTCCCG |
| FHal108 | C7QKD3 | FAD dependent oxidoreductase | *Catenulispora acidiphila (strain DSM 44928 / NRRL B-24433 / NBRC 102108 / JCM 14897)* | DSMZ-44928 | AAAGAAGGAGATAGGATCATGAGCGAAGACCGTTATGACGTG | GTGTAATGGATAGTGATCTTATGCGGTGATCTCCTGGACCGCCTG |
| FHal109 | D4XB54 | FAD dependent oxidoreductase | *Achromobacter piechaudii ATCC 43553* | DSMZ-10342 | AAAGAAGGAGATAGGATCATGGAACATCGTGAAGTCGTCGTC | GTGTAATGGATAGTGATCTTAAGCGGGTTCGGCCTGCGCG |
| FHal110 | D6CVB6 | Putative oxidoreductase, FAD-binding | *Thiomonas arsenitoxydans (strain DSM 22701 / CIP 110005 / 3As)* | DSMZ-22701 | AAAGAAGGAGATAGGATCATGACAGAACGACAGATTGTGTCTGGCGAC | GTGTAATGGATAGTGATCTTAGCCGTGGTGAGTCTCAGGCTGGGG |
| FHal111 | D7BIJ6 | 4-hydroxyphenylacetate 3-monooxygenase, oxygenase subunit | *Meiothermus silvanus (strain ATCC 700542 / DSM 9946 / VI-R2)* | DSMZ-9946 | AAAGAAGGAGATAGGATCATGGCACGCACCGGCAAACAGTACCTC | GTGTAATGGATAGTGATCTTAGTCCCCCGCTATCACTTCGGGTTGTGG |
| FHal112 | E1ST92 | Tryptophan halogenase | *Ferrimonas balearica (strain DSM 9799 / CCM 4581 / PAT)* | DSMZ-9799 | AAAGAAGGAGATAGGATCATGACTCAACCGCTTAATCGCATCA | GTGTAATGGATAGTGATCTTAGTTCAGGGCACGCAGTGCCG |
| FHal113 | E1SVG0 | Tryptophan halogenase | *Ferrimonas balearica (strain DSM 9799 / CCM 4581 / PAT)* | DSMZ-9799 | AAAGAAGGAGATAGGATCATGCAGCGACAACAGGTGGTGATCCTCG | GTGTAATGGATAGTGATCTTAGGGGGTGTTCGGCATCGGGC |
| FHal114 | E8RKM3 | Tryptophan halogenase | *Asticcacaulis excentricus (strain ATCC 15261 / DSM 4724 / VKM B-1370 / CB 48)* | DSM-4724 | AAAGAAGGAGATAGGATCATGAGCGACGCCATTCGCCGTATTATCATC | GTGTAATGGATAGTGATCTTAGACGCTGGCTTTCAGTGTATCGGGCG |
| FHal115 | E8RRR9 | Tryptophan halogenase | *Asticcacaulis excentricus (strain ATCC 15261 / DSM 4724 / VKM B-1370 / CB 48)* | DSM-4724 | AAAGAAGGAGATAGGATCATGCAACCGGTCAAAAAGGTAGTTAT | GTGTAATGGATAGTGATCTTACGCCGCCAGATAGCTTTTGAGGAAGGC |
| FHal116 | E8RTM7 | Tryptophan halogenase | *Asticcacaulis excentricus (strain ATCC 15261 / DSM 4724 / VKM B-1370 / CB 48)* | DSM-4724 | AAAGAAGGAGATAGGATCATGGCGAAGCGCCCTTACTCTTCCA | GTGTAATGGATAGTGATCTTAAGCGGTCCCTGCCTCTGCCTGG |
| FHal117 | E8U8Z0 | 4-hydroxyphenylacetate 3-monooxygenase, oxygenase subunit | *Deinococcus maricopensis (strain DSM 21211 / LMG 22137 / NRRL B-23946 / LB-34)* | DSM-21211 | AAAGAAGGAGATAGGATCATGGGCGCACGTAATGGTCAGGCCTTC | GTGTAATGGATAGTGATCTTAGTCATCGGCGCCCACCAGTTCCG |
| FHal118 | F4CSY5 | 4-hydroxyphenylacetate 3-hydroxylase | *Pseudonocardia dioxanivorans (strain ATCC 55486 / DSM 44775 / JCM 13855 / CB1190)* | DSMZ-44775 | AAAGAAGGAGATAGGATCATGACCACCACGAAGGAACCTGC | GTGTAATGGATAGTGATCTTAGGGTGTTCCGATGCGCTTCC |
| FHal119 | H6N3Z6 | Putative tryptophan halogenase | *Gordonia polyisoprenivorans (strain DSM 44266 / VH2)* | DSMZ-44266 | AAAGAAGGAGATAGGATCATGTATGACGTCATTGTGGTAGGCG | GTGTAATGGATAGTGATCTTATGCCGGCACCAGATGCCGG |
| FHal120 | H6R579 | Aromatic ring hydroxylase | *Nocardia cyriacigeorgica (strain GUH-2)* | INTERNE | AAAGAAGGAGATAGGATCATGACACCGAAAGCTGCCCCCACCAC | GTGTAATGGATAGTGATCTTACCGGCGACGCCAGTCACGA |
| FHal121 | I0H626 | NAD(FAD)-dependent dehydrogenase | *Actinoplanes missouriensis (strain ATCC 14538 / DSM 43046 / CBS 188.64 / JCM 3121 / NCIMB 12654 / NBRC 102363 / 431)* | DSMZ-43046 | AAAGAAGGAGATAGGATCATGAACTCCGAGAGCTACGACGTC | GTGTAATGGATAGTGATCTTATGGTCTGGTCCAGCCGAGGCTGTCG |
| FHal122 | J0L214 | Uncharacterized protein | *Rhizobium leguminosarum bv. trifolii WSM2297* | DSMZ-6040 | AAAGAAGGAGATAGGATCATGCGATCAGAAGATTTCCGGGCCGATAC | GTGTAATGGATAGTGATCTTATTCAGCAGCGGACTTGAACTGG |
| FHal123 | K9D7H2 | Halogenase | *Sphingobium yanoikuyae ATCC 51230* | DSMZ-7462 | AAAGAAGGAGATAGGATCATGCATGCAGATTTTCCGACGTCCGACCG | GTGTAATGGATAGTGATCTTAGACCAATTCTGCGGGACAATAGCGGCG |
| FHal124 | L0A0B2 | FAD-binding monooxygenase | *Deinococcus peraridilitoris (strain DSM 19664 / LMG 22246 / CIP 109416 / KR-200)* | DSMZ-19664 | AAAGAAGGAGATAGGATCATGGGAGCACGAACCGGACAACAG | GTGTAATGGATAGTGATCTTAATCGTCCGCCGCGACGCCCTG |
| FHal125 | Q0RGW0 | Putative Halogenase | *Frankia alni (strain ACN14a)* | INTERNE | AAAGAAGGAGATAGGATCATGGAGAATTCCACGCAGGTTCTTG | GTGTAATGGATAGTGATCTTACTGGAGGTTGGCGAGCCCC |
| FHal126 | Q0RMQ6 | Tryptophan halogenase | *Frankia alni (strain ACN14a)* | INTERNE | AAAGAAGGAGATAGGATCATGCTGTTCGCCCGGCAGGGATACCG | GTGTAATGGATAGTGATCTTAGCTCTGGTCGAGCACGGCG |
| FHal127 | Q0RS73 | Tryptophan halogenase | *Frankia alni (strain ACN14a)* | INTERNE | AAAGAAGGAGATAGGATCATGAGCGGGTACGACTATTCGGTTGCCATC | GTGTAATGGATAGTGATCTTAGAAGACGGCCGGTTGCGGCG |
| FHal128 | Q1GUN6 | Monooxygenase, FAD-binding:FAD dependent oxidoreductase:Tryptophan halogenase | *Sphingopyxis alaskensis (strain DSM 13593 / LMG 18877 / RB2256)* | DSMZ-13593 | AAAGAAGGAGATAGGATCATGACGGGCATGGACAGCAGCACC | GTGTAATGGATAGTGATCTTATGCGTCGTTCCGCCCGCCG |
| FHal129 | Q1GUN9 | FAD dependent oxidoreductase | *Sphingopyxis alaskensis (strain DSM 13593 / LMG 18877 / RB2256)* | DSMZ-13593 | AAAGAAGGAGATAGGATCATGGGCACGTCGATTCATAATGTGGTGAT | GTGTAATGGATAGTGATCTTAGGCGGCGTGCGCCCGAAGTG |
| FHal130 | Q1QTV5 | FAD dependent oxidoreductase | *Chromohalobacter salexigens (strain DSM 3043 / ATCC BAA-138 / NCIMB 13768)* | DSMZ-3043 | AAAGAAGGAGATAGGATCATGAACCCAAGCGTCCCAGACTG | GTGTAATGGATAGTGATCTTATGAGCGTACCTCGTCGCGGT |
| FHal131 | Q2G7X6 | Pyoverdin chromophore biosynthetic protein PvcC | *Novosphingobium aromaticivorans (strain DSM 12444 / F199)* | DSMZ-12444 | AAAGAAGGAGATAGGATCATGAAGCGTCCGAACCCCGCAGTCC | GTGTAATGGATAGTGATCTTATGCAGCCGCCGGCATTTCG |
| FHal132 | Q2T8R6 | 4-hydroxyphenylacetate 3-monooxygenase | *Burkholderia thailandensis (strain E264 / ATCC 700388 / DSM 13276 / CIP 106301)* | DSMZ-13276 | AAAGAAGGAGATAGGATCATGACGTTGATGACGGGAAACGAC | GTGTAATGGATAGTGATCTTAGGGTTCGAGCGGCGTGCTTCG |
| FHal133 | Q8P3Q6 | Tryptophan halogenase | *Xanthomonas campestris pv. campestris (strain ATCC 33913 / NCPPB 528 / LMG 568)* | DSMZ-3586 | AAAGAAGGAGATAGGATCATGCCGGCAGCCGGGCAGGC | GTGTAATGGATAGTGATCTTATGGAGCGTCTCTGTGCAAGGT |
| FHal134 | Q8P6Z0 | Tryptophan halogenase, putative | *Xanthomonas campestris pv. campestris (strain ATCC 33913 / NCPPB 528 / LMG 568)* | DSMZ-3586 | AAAGAAGGAGATAGGATCATGACGCAGGCACGCATTCGCAAG | GTGTAATGGATAGTGATCTTAGCCCCAGGTGCTTTGATCCAC |
| FHal135 | Q9A4M5 | Tryptophan halogenase, putative | *Caulobacter crescentus (strain ATCC 19089 / CB15)* | DSMZ-4727 | AAAGAAGGAGATAGGATCATGGACCCTATCCGCAAGATCGTCATCGTC | GTGTAATGGATAGTGATCTTATGCCGGCGTCCTCGCATCGG |
| FHal136 | Q9A7G8 | Tryptophan halogenase, putative | *Caulobacter crescentus (strain ATCC 19089 / CB15)* | DSMZ-4727 | AAAGAAGGAGATAGGATCATGCAACCGCTCAAAAAGATTCTGATCGCC | GTGTAATGGATAGTGATCTTATGAGGTGGGAACAGGCGCGAGCGG |
| FHal137 | Q9A835 | Tryptophan halogenase, putative | *Caulobacter crescentus (strain ATCC 19089 / CB15)* | DSMZ-4727 | AAAGAAGGAGATAGGATCATGGGCGAGGCGATTCAGTCGATTGTT | GTGTAATGGATAGTGATCTTAGGCCTCGGGTTCCGCCCAGG |
| FHal138 | Q9A9H8 | Tryptophan halogenase | *Caulobacter crescentus (strain ATCC 19089 / CB15)* | DSMZ-4727 | AAAGAAGGAGATAGGATCATGCAACCCCAAACGATCCGCAAGG | GTGTAATGGATAGTGATCTTAGGCGCTCTTGCTGACCGCC |
| FHal139 | Q9A9K8 | Uncharacterized protein | *Caulobacter crescentus (strain ATCC 19089 / CB15)* | DSMZ-4727 | AAAGAAGGAGATAGGATCATGACCACCACCCACCCCAACG | GTGTAATGGATAGTGATCTTAAGCCACCGCCCCAAACGGCG |
| FHal140 | S5V8T9 | Tryptophan halogenase | *Streptomyces collinus Tu 365* | DSMZ-40129 | AAAGAAGGAGATAGGATCATGACCGACAACGACTCGCGTATC | GTGTAATGGATAGTGATCTTACTTGCCGTGCAGCCGGCGC |
| FHal141 | S9NY11 | Tryptophan halogenase, putative | *Cystobacter fuscus DSM 2262* | DSMZ-2262 | AAAGAAGGAGATAGGATCATGAGCACCATGCGACCCACCCC | GTGTAATGGATAGTGATCTTAGCCGCGTGGGCTGGAGATGAGC |
| FHal142 | S9QUE7 | Tryptophan halogenase, putative | *Rubellimicrobium thermophilum DSM 16684* | DSM-16684 | AAAGAAGGAGATAGGATCATGACGGATCGCGGCTTCCGCCC | GTGTAATGGATAGTGATCTTACCCGCCCCCCGCTTCGAG |
| FHal143 | U7DT88 | Uncharacterized protein | *Pseudomonas fluorescens NCIMB 11764* | DSMZ-50090 | AAAGAAGGAGATAGGATCATGGAAAATAACGCAAACAAAAAACCG | GTGTAATGGATAGTGATCTTATTGGCCGTTGATGCGCTGATC |
| FHal144 | V4PHG1 | Uncharacterized protein | *Asticcacaulis benevestitus DSM 16100 = ATCC BAA-896* | DSMZ-16100 | AAAGAAGGAGATAGGATCATGACGGCGGCAGCGCTTGCCCATA | GTGTAATGGATAGTGATCTTAACCCACGCCTGCGGTGAGCG |
| FHal145 | V4PP63 | Tryptophan halogenase | *Asticcacaulis benevestitus DSM 16100 = ATCC BAA-896* | DSMZ-16100 | AAAGAAGGAGATAGGATCATGACACGCAGCGCCGATCATTTTGTC | GTGTAATGGATAGTGATCTTAAACCGGTGACAAAGGCCGCCTGAGC |
| FHal146 | B2GI35 | Putative oxidoreductase | *Kocuria rhizophila (strain ATCC 9341 / DSM 348 / NBRC 103217 / DC2201)* | DSMZ-348 | AAAGAAGGAGATAGGATCATGAAGGTAGTGATCAGCGGGGGTGGCCC | GTGTAATGGATAGTGATCTTAGCGCGCGGGTGTCAGCG |
| FHal147 | Q7NSZ6 | Tryptophan halogenase | *Chromobacterium violaceum (strain ATCC 12472 / DSM 30191 / JCM 1249 / NBRC 12614 / NCIMB 9131 / NCTC 9757)* | DSMZ-30191 | AAAGAAGGAGATAGGATCATGAGCGTTAGCCGCTACCCGGTG | GTGTAATGGATAGTGATCTTAGGCGGGAACGGCCAGCG |
| FHal148 | S9P988 | Tryptophan halogenase | *Cystobacter fuscus DSM 2262* | DSMZ-2262 | AAAGAAGGAGATAGGATCATGGACACCCCTGACTTCGAGGCCC | GTGTAATGGATAGTGATCTTATGCCGCCACGGACGTGCCCAG |

Table S3. Kinetic parameters of FHals for which tryptophan is the metabolic substrate; comparison with the kinetic parameters of *Xszen*FHal

| Enzyme | *k*_cat_ min^-1^ | *K*_m_ mM | *k*_cat_/*K*_m_ | reference |
| --- | --- | --- | --- | --- |
| Stth | 1,53 | 21 | 0,073 | Zeng et al. Biotechnol. Lett. 2011, 33, 1607 |
| Tar14 | 0,42 | 12 | 0,035 | Luhavaya et al. Ang. Chem. Int. Ed. 2019, 58, 8394-8399 |
| KtzQ | 0,19 | ≤2 | ≥0,095 | Heemstra et al. JACS 2008, 130, 14024 |
| PrnA | 0,093 | 160 | 0,001 | Dong et al. Science, 2005, 309, 2216-2219 |
| RebH | 1,4 | 2 | 0,700 | Yeh et al. PNAS 2005, 102, 3960 |
| PyrH | 3,56 | 109 | 0,033 | Zhu et al. J.Mol.Biol. 2009, 391, 75-85 |
| ***Xszen*FHal** | **4,44** | **58,22** | **0,076** |  |

**Figures**

Figure S1. Percent identity matrix of the reference set


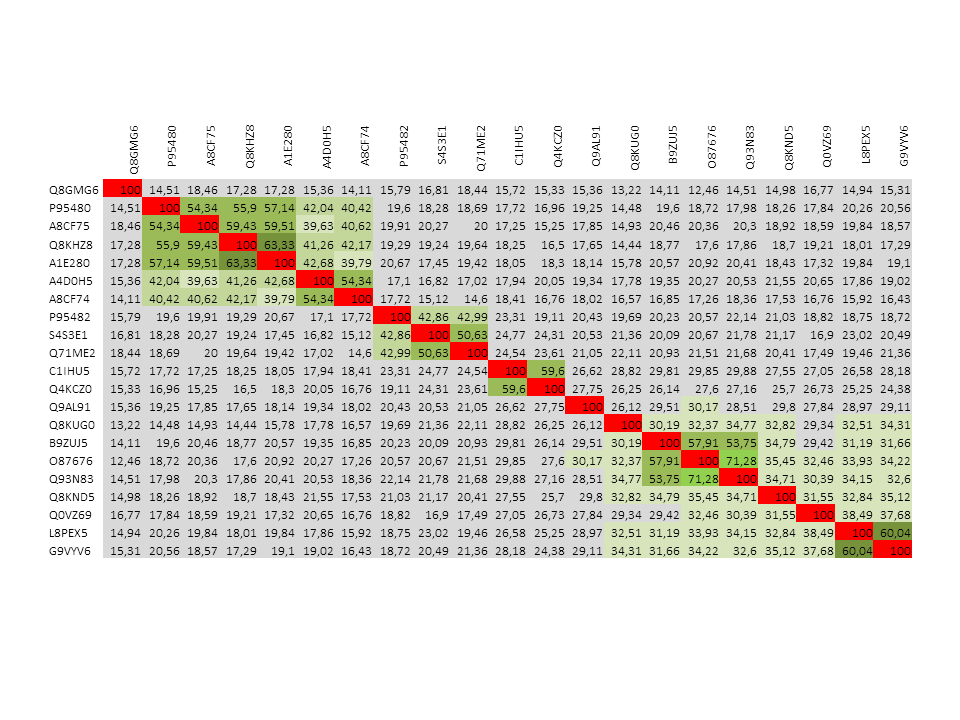


Figure S2. UHPLC traces of substrates and their corresponding chlorinated derivatives


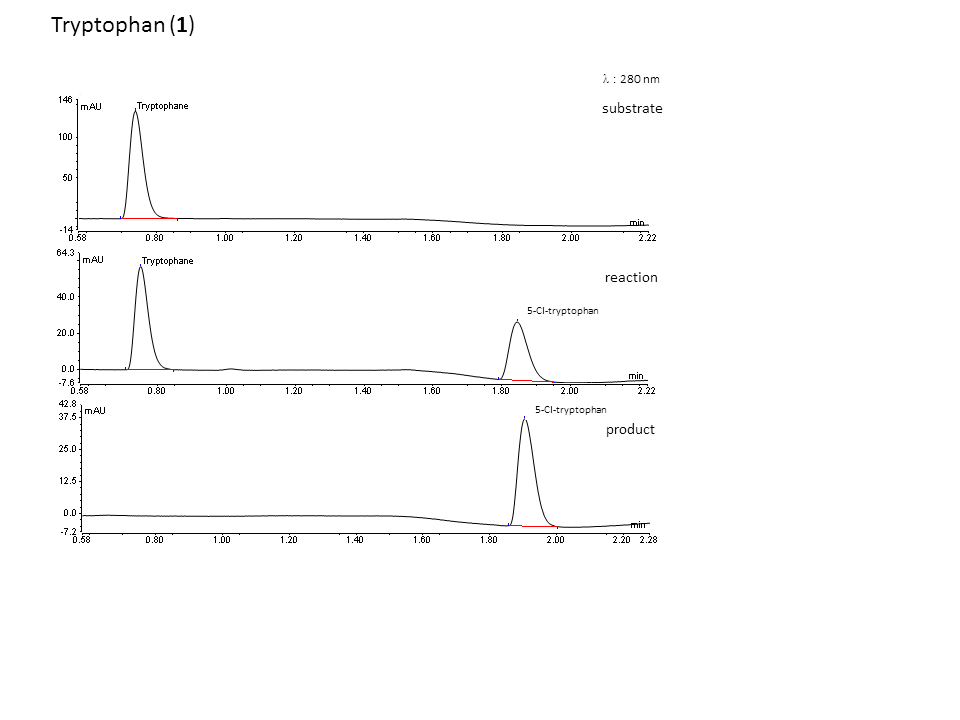


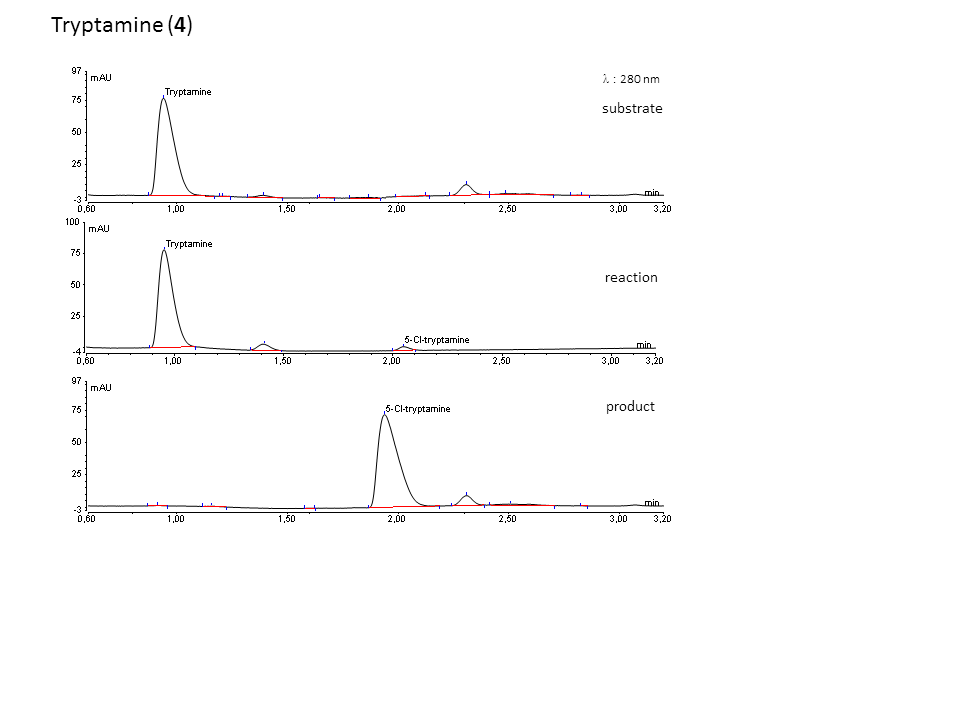


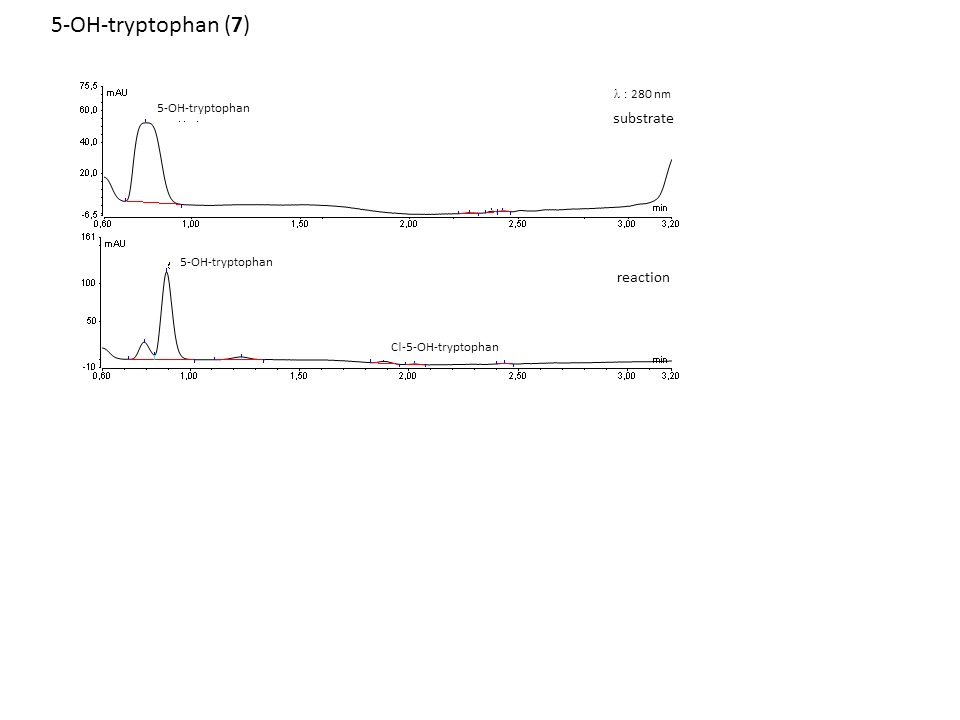


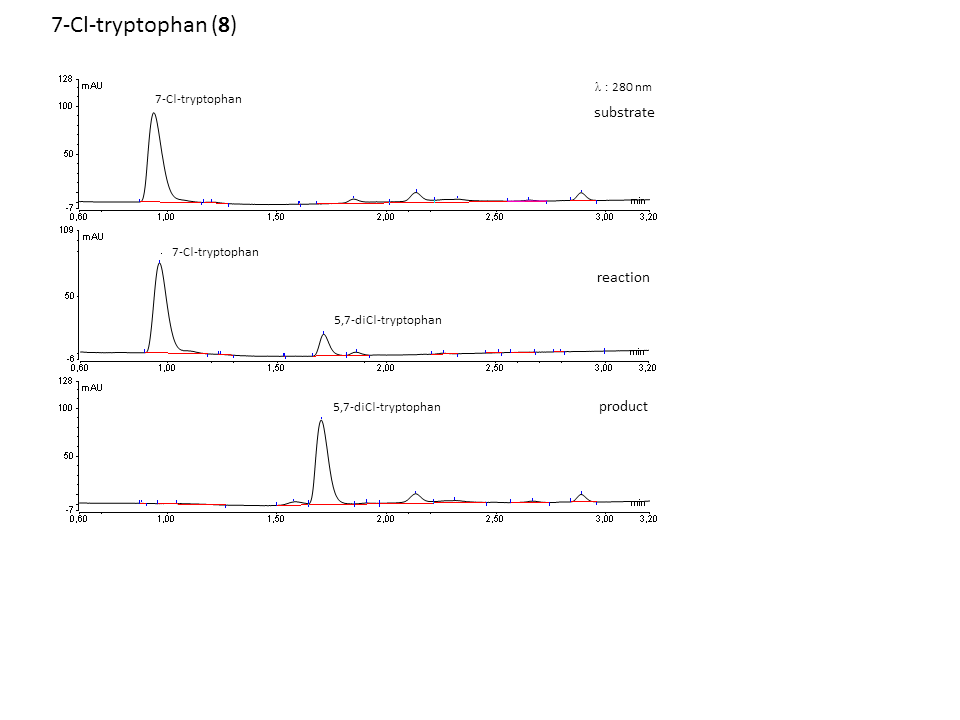


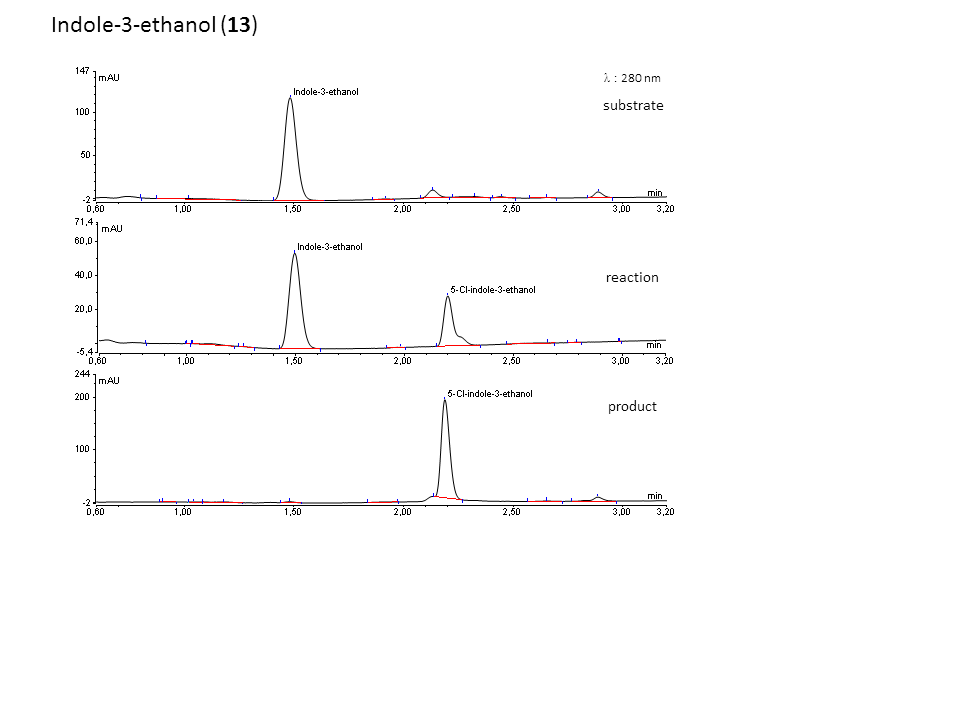


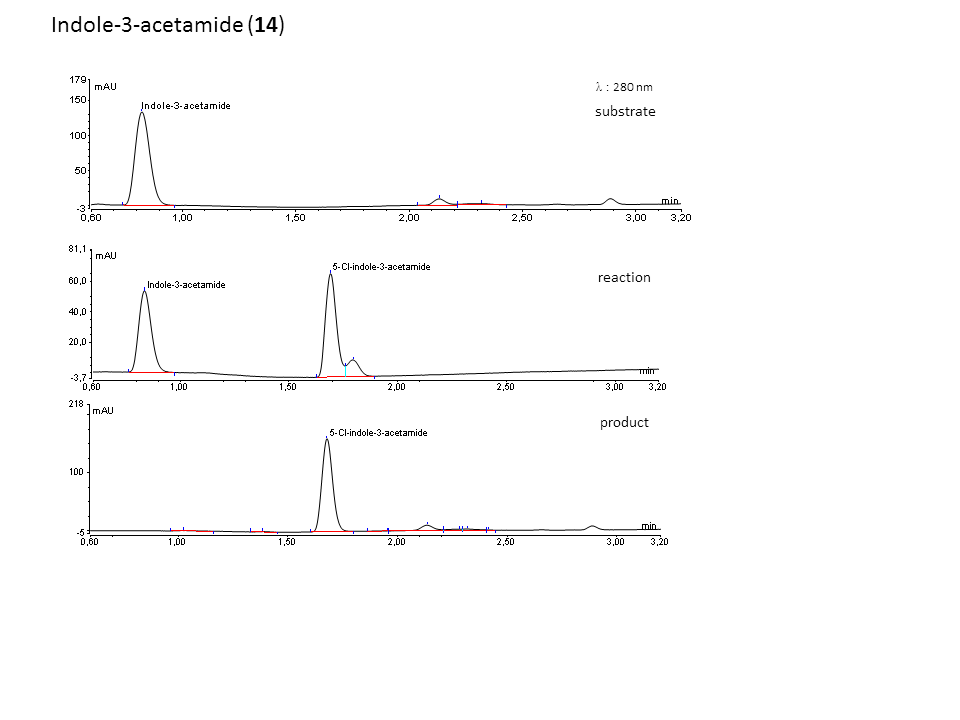


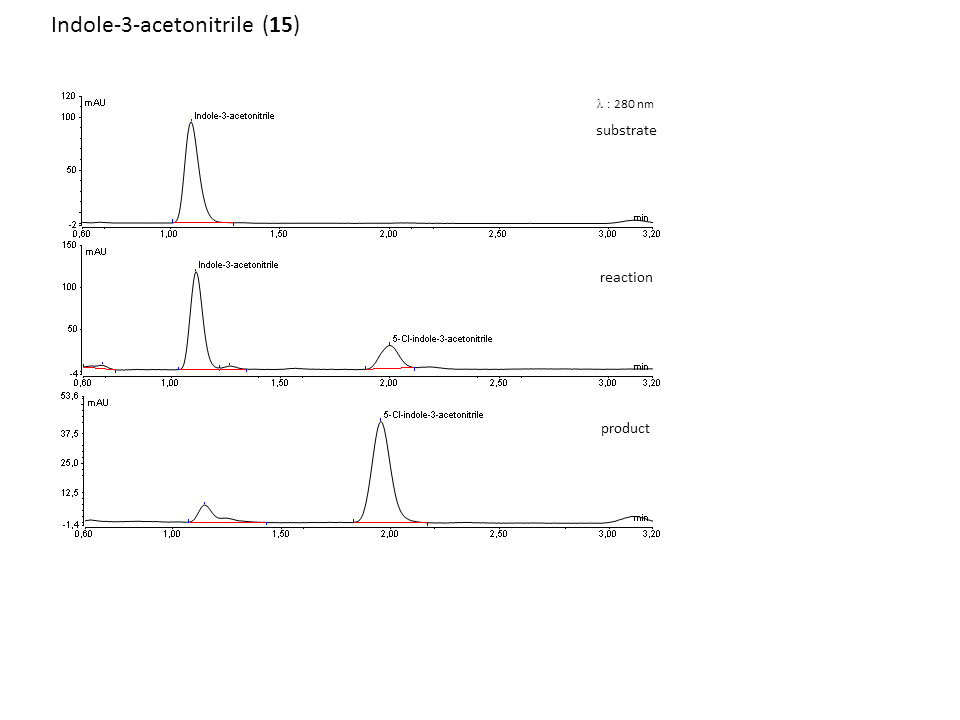


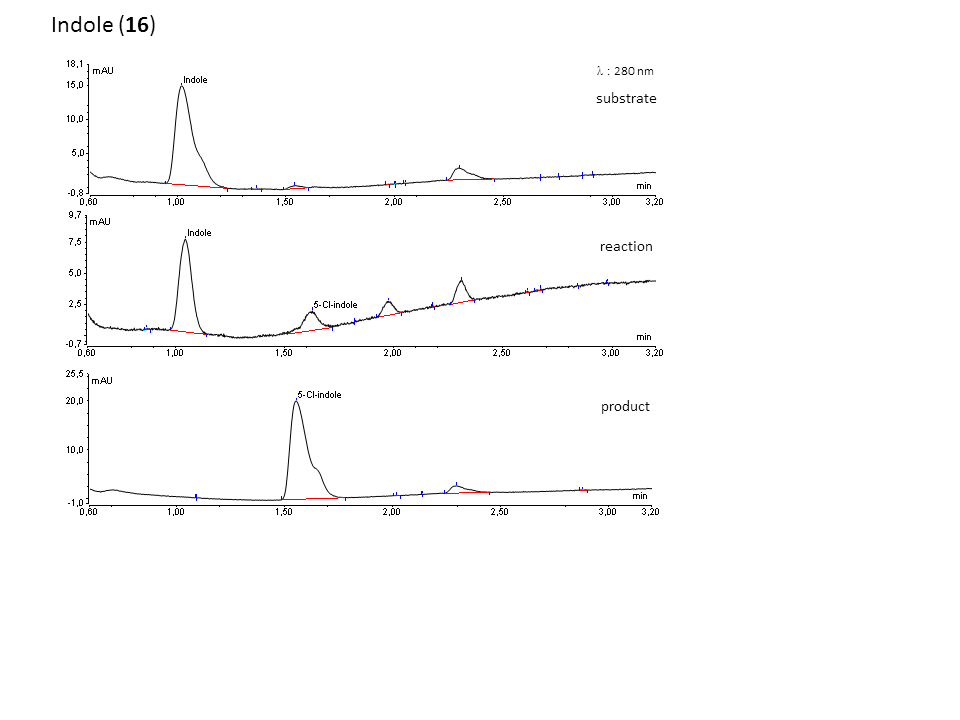


Figure S3. MS spectra of 5-chlorotryptophan.


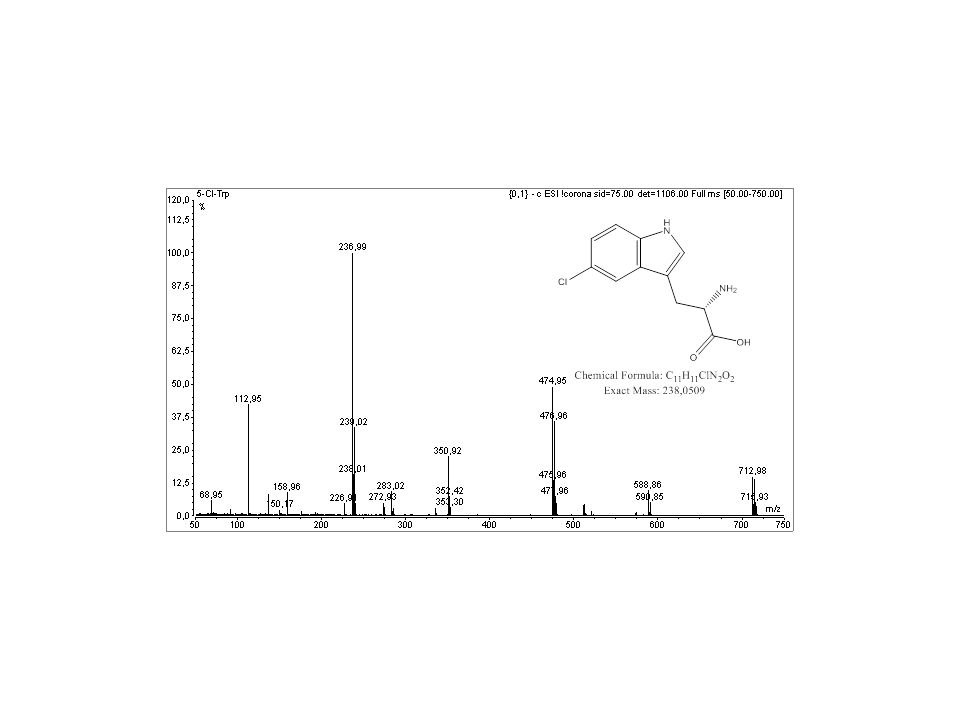


Figure S4. Structures of the pyrroindomycin B, an antibiotic compound produced by *Streptomyces rugosporus* LL-42D005, and metabolites from *Xenorhabdus szentirmaii*. The tryptophan moieties are highlighted in red ([Ohlendorf et al. 2011](#_ENREF_2); [Wenski et al. 2019](#_ENREF_6); [Zehner et al. 2005](#_ENREF_7))

Figure S5. **A**. UHPLC trace of the tryptophan bromination reaction by *Xszen*FHal. **B**. Time course of the conversion of tryptophan by *Xszen*FHal with NaBr over time


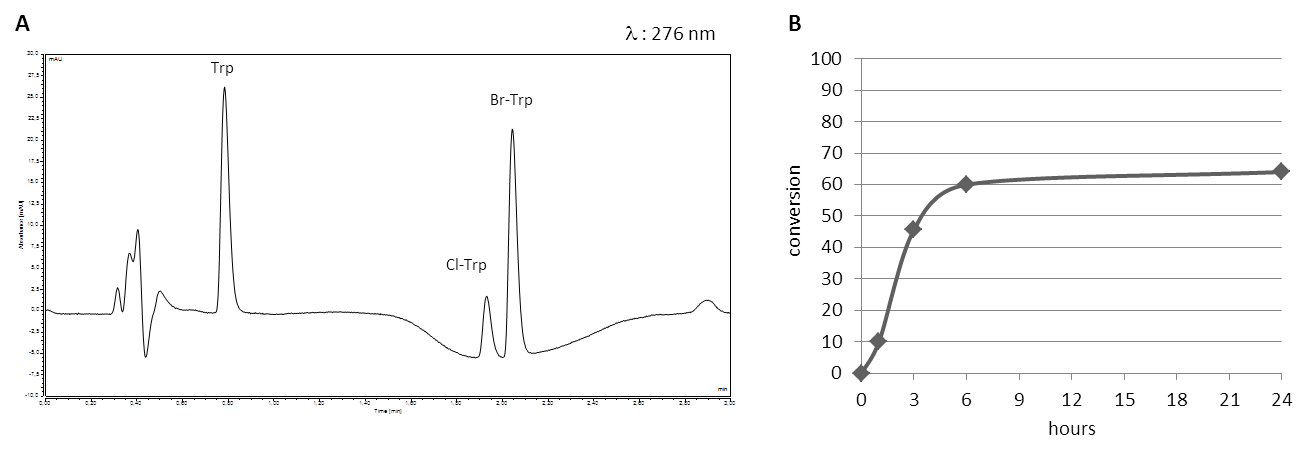


Reaction conditions. 1 mM L-tryptophan, 0.25 mg/mL *Xszen*FHal, 0.055 mg/mL *K12*Fre, 20 mM NaX, 3 mM glucose, 1 U/mL GDH, 0.025 mM NADH, 1 μM FAD in phosphate buffer 10 mM pH 7.4. The reaction was stirred at 500 rpm for 24 h then 1 mM TFA and 70 mL isopropanol were added. After centrifugation at 6000 rpm for 10 min, the supernatant was filtered over PVDF membrane filter 0.2 mM and analyzed by UHPLC-UV using the following conditions: linear gradient (ratio A/B 99/1 during 1 min, then 99/1 to 85/15 in 2.5 min, 85/15 for 2.5 min then 85/15 to 50/50 in 1 min), flow 0.4 mL/min, λ = 276 nm.

Figure S6. Plots for determination of kinetic parameters of *Xszen*FHal. **A**. Determination of the initial velocity. **B**. Michaelis-Menten kinetics


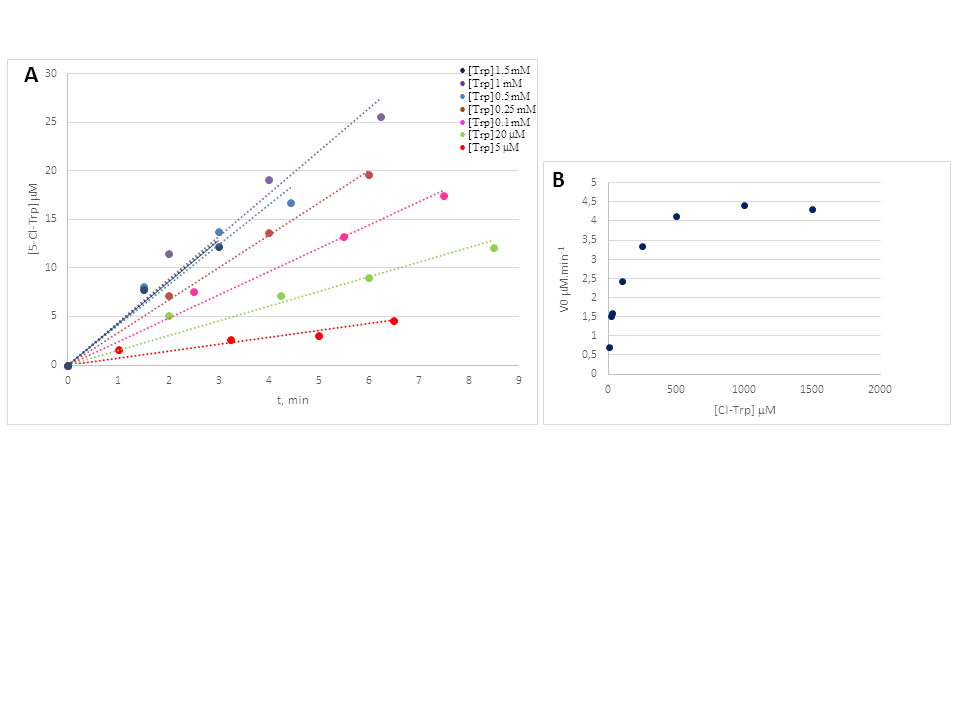


Reaction conditions. 1 μM *Xszen*FHal, 0.1 μM *K12*Fre, 20 mM NaCl, 0.05 mM NADH, 0.01 mM FAD in phosphate buffer 10 mM pH 7.5. The following tryptophan concentrations were used: 5 μM; 20 μM; 100 μM; 250 μM; 500 μM; 1000 μM; 1500 μM. The reaction was stirred at 500 rpm then 1 μM TFA and 70 μL isopropanol were added. After centrifugation at 6000 rpm for 10 min, the supernatant was filtered over PVDF membrane filter 0.2 μm and analyzed by UHPLC-UV.

Figure S7. Percent identity matrix of the sequences of the 5-tryptophan halogenases ([Ortega et al. 2017](#_ENREF_3); [Son et al. 2017](#_ENREF_5); [Zehner et al. 2005](#_ENREF_7); [Zhu et al. 2009](#_ENREF_8))

|  | W2EQU4 | A0A2D3E318 | A4D0H5 | W1J423 |
| --- | --- | --- | --- | --- |
| W2EQU4 (MlbH) | 100 | 34 | 37 | 31 |
| A0A2D3E318 (Ulm24) | 34 | 100 | 58 | 54 |
| A4D0H5 (PyrH) | 37 | 58 | 100 | 60 |
| W1J423 (*Xszen*FHal) | 31 | 54 | 60 | 100 |

**References**

Heemstra JR, Walsh CT (2008) Tandem Action of the O2- and FADH2-Dependent Halogenases KtzQ and KtzR Produce 6,7-Dichlorotryptophan for Kutzneride Assembly. J Am Chem Soc 130(43):14024-14025 doi:10.1021/ja806467a

Ohlendorf B, Simon S, Wiese J, Imhoff JF (2011) Szentiamide, an N-formylated Cyclic Depsipeptide from *Xenorhabdus szentirmaii* DSM 16338T. Nat Prod Commun 6(9):1934578X1100600909 doi:10.1177/1934578x1100600909

Ortega MA, Cogan DP, Mukherjee S, Garg N, Li B, Thibodeaux GN, Maffioli SI, Donadio S, Sosio M, Escano J, Smith L, Nair SK, van der Donk WA (2017) Two Flavoenzymes Catalyze the Post-Translational Generation of 5-Chlorotryptophan and 2-Aminovinyl-Cysteine during NAI-107 Biosynthesis. ACS Chem Biol 12(2):548-557 doi:10.1021/acschembio.6b01031

Podzelinska K, Latimer R, Bhattacharya A, Vining LC, Zechel DL, Jia Z (2010) Chloramphenicol Biosynthesis: The Structure of CmlS, a Flavin-Dependent Halogenase Showing a Covalent Flavin–Aspartate Bond. J Mol Biol 397(1):316-331 doi:10.1016/j.jmb.2010.01.020

Son S, Hong Y-S, Jang M, Heo KT, Lee B, Jang J-P, Kim J-W, Ryoo I-J, Kim W-G, Ko S-K, Kim BY, Jang J-H, Ahn JS (2017) Genomics-Driven Discovery of Chlorinated Cyclic Hexapeptides Ulleungmycins A and B from a *Streptomyces* Species. J Nat Prod 80(11):3025-3031 doi:10.1021/acs.jnatprod.7b00660

Wenski SL, Kolbert D, Grammbitter GLC, Bode HB (2019) Fabclavine biosynthesis in *X. szentirmaii*: shortened derivatives and characterization of the thioester reductase FclG and the condensation domain-like protein FclL. J Ind Microbiol Biotechnol 46(3):565-572 doi:10.1007/s10295-018-02124-8

Zehner S, Kotzsch A, Bister B, Sussmuth RD, Mendez C, Salas JA, van Pee KH (2005) A regioselective tryptophan 5-halogenase is involved in pyrroindomycin biosynthesis in *Streptomyces rugosporus* LL-42D005. Chem Biol 12(4):445-52 doi:10.1016/j.chembiol.2005.02.005

Zhu X, De Laurentis W, Leang K, Herrmann J, Ihlefeld K, van Pée K-H, Naismith JH (2009) Structural Insights into Regioselectivity in the Enzymatic Chlorination of Tryptophan. J Mol Biol 391(1):74-85 doi:10.1016/j.jmb.2009.06.008

1. Génomique métabolique, Genoscope, Institut François Jacob, CEA, CNRS, Univ Evry, Univ Paris-Saclay, 91057 Evry, France

   ^┴^ These authors have contributed equally to this work [↑](#footnote-ref-1)
